# Middle Stone Age human teeth from Magubike rockshelter, Iringa Region, Tanzania

**DOI:** 10.1371/journal.pone.0200530

**Published:** 2018-07-31

**Authors:** Pamela R. Willoughby, Tim Compton, Silvia M. Bello, Pastory M. Bushozi, Anne R. Skinner, Chris B. Stringer

**Affiliations:** 1 Department of Anthropology, University of Alberta, Edmonton, Alberta, Canada; 2 Department of Earth Sciences, Natural History Museum, London, United Kingdom; 3 Department of History and Archaeology, College of Humanities, University of Dar es Salaam, Dar es Salaam, Tanzania; 4 Department of Chemistry, Williams College, Williamstown, Massachusetts, United States of America; Université de Poitiers, FRANCE

## Abstract

In 2006, six isolated hominin teeth were excavated from Middle Stone Age (MSA) deposits at the Magubike rockshelter in southern Tanzania. They comprise two central incisors, one lateral incisor, one canine, one third premolar, and one fourth premolar. All are fully developed and come from the maxilla. None of the teeth are duplicated, so they may represent a single individual. While there is some evidence of post-depositional alteration, the morphology of these teeth clearly shares features with anatomically modern *Homo sapiens*. Both metric and non-metric traits are compared to those from other African and non-African dental remains. The degree of biological relatedness between eastern and southern African Stone Age hunter-gatherers has long been a subject of interest, and several characteristics of the Magubike teeth resemble those of the San of southern Africa. Another notable feature is that the three incisors are marked on the labial crown by scratches that are much coarser than microwear striations. These non-masticatory scratches on the Magubike teeth suggest that the use of the front teeth as tools included regularly repeated activities undertaken throughout the life of the individual. The exact age of these teeth is not clear as ESR and radiocarbon dates on associated snail shells give varying results, but a conservative estimate of their minimum age is 45,000 years.

## Introduction

The earliest *Homo sapiens* skeletal remains showing a modern-looking cranial shape come from the continent of Africa and are dated between 150,000 and 200,000 years ago [127–128]. However, before and during this period, there is also evidence of populations attributable to the lineage of *Homo sapiens* that lack a predominance of the derived features found in anatomically modern humans, and that we will term here archaic *Homo sapiens* (examples of such specimens include those from Jebel Irhoud and Ngaloba, Laetoli [1–2]. Note that in our usage, hominin remains from the Mugharet es-Skhūl and Jebel Qafzeh represent early anatomically modern *Homo sapiens*, not archaic *sapiens*. In Africa, *Homo heidelbergensis*, archaic *Homo sapiens*, and anatomically modern *Homo sapiens* remains have all been recovered in actual or inferred association with Middle Stone Age (MSA) artifacts at a number of localities [3]. MSA occupations are dominated by flake tools made on radial or discoidal cores; some of these are retouched into tools, while others are manufactured using the Levallois technology. The objective was to produce points and/or scrapers, many of which were evidently hafted onto organic handles.

**Fig 1:**
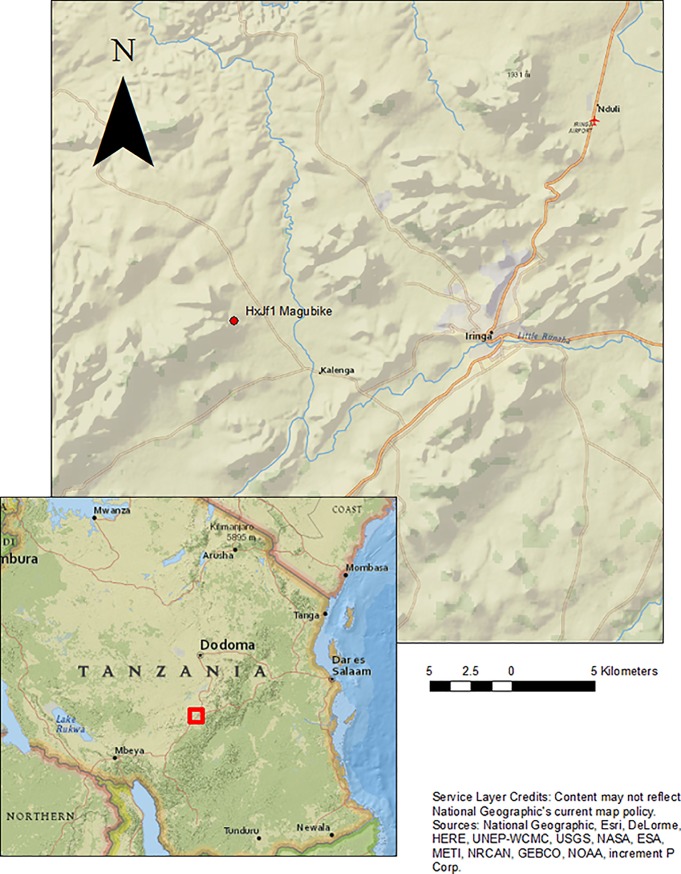
Magubike rockshelter, Iringa Region, southern Tanzania (map produced by Jeffrey Werner).

This paper presents results of the analysis of several teeth that come from a new archaeological site, Magubike rockshelter, located in the southern highlands of Tanzania (Fig 1). Magubike has extensive MSA and later cultural deposits (Later Stone Age or LSA, Iron Age and recent/historic occupation levels). It is located in Iringa Rural (*Iringa Vijijini* in Kiswahili), a district in Iringa Region in the southern Highlands of Tanzania. The landscape of Iringa is dominated by Precambrian highlands, and many rockshelters and inselbergs can be observed.

**Fig 2:**
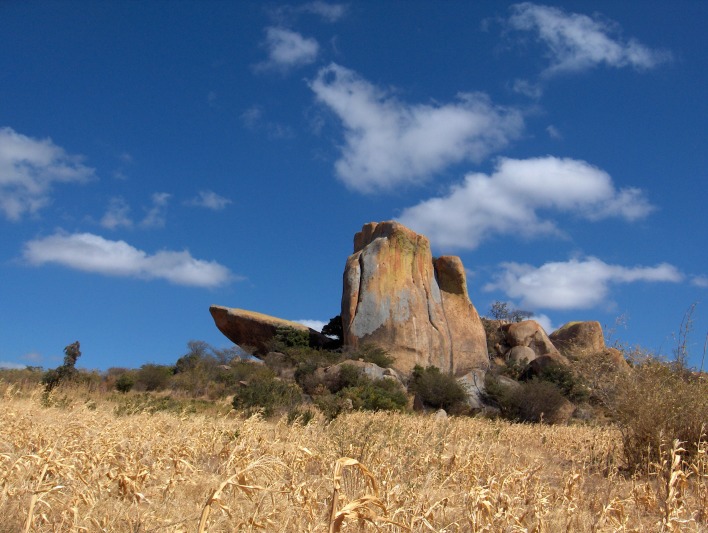
Photograph of Magubike rockshelter.

The archaeological site of Magubike is a large granite rockshelter on a hill overlooking the modern village of the same name. It is located at 7°45.790’S, 35°28.399’E, at an elevation of 1541 m (Fig 2). Pamela Willoughby was shown this rockshelter in June 2005 as part of an initial reconnaissance of the area north of Iringa City on the road to the Ruaha National Park [4]. It was recorded as SASES (Standardized African Site Enumeration System) site # HxJf-01 and on the surface there were LSA (Later Stone Age) quartz and crypto-crystalline artifacts, as well as Iron Age pottery and iron pieces and iron slag. Bone and shell were also present. At an elevation of 1,666 m, in fallow maize and tobacco fields, one could also see numerous MSA artifacts.

The members of the Iringa Region Archaeological Project (IRAP) conducted initial test excavations at Magubike rockshelter in August 2006. The goal was to locate stratified MSA deposits in direct association with organic remains and datable material. Up until this time, the only Stone Age site studied in any detail in Iringa was the Isimila Korongo, an Acheulean (and later) locality first excavated in the 1950s [5]. Korongo is gully in Kiswahili and is usually added to the site name to distinguish it from nearby places with the same name.

## Geological / Archaeological context

**Fig 3:**
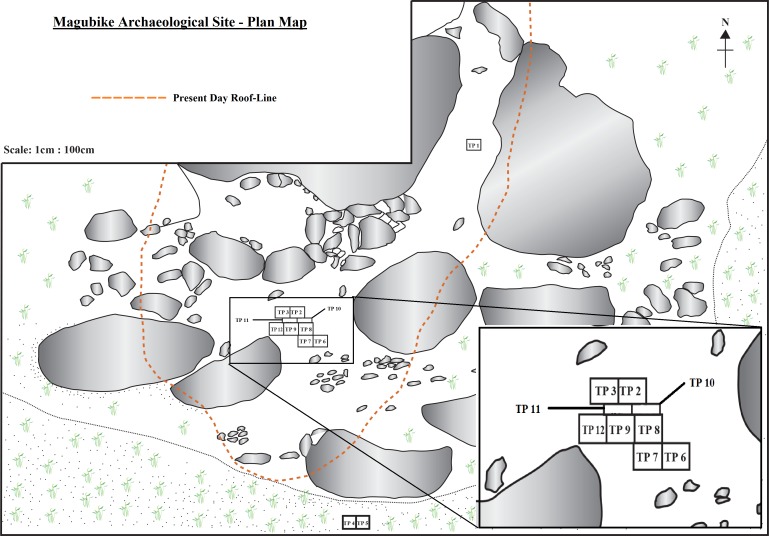
Plan view of excavations at Magubike. Previously published as Werner and Willoughby (2017:252).

In 2006, three test pits of 1 metre square were dug in 10 cm artificial levels at Magubike. Test pit 1 was in a side chamber and produced a cultural sequence of historic, Iron Age, LSA artifacts, and then, below 40 cm of sediment with very few artifacts, a MSA deposit was encountered that extended from 110 to 180 cm depth, where bedrock was reached. Organic materials were only present in the historic and Iron Age layers. Two additional square metres were excavated in the main shelter, under the modern overhang (Fig 3). The first, test pit 2, contained historic and Iron Age materials in the top 50 cm, and then an entirely different MSA deposit was encountered. While the MSA artifacts in test pit 1 were dominated by quartz and light-coloured chert artifacts, those in test pit 2 were dark metamorphic rocks and yellow cherts [6–7]. Further excavation was impeded by a large piece of roof fall, but one strip, about 15 cm wide, remained at the west side. Consequently, this excavation was extended 1 metre to the west (test pit 3). Test pit 3 extended from the surface to 210 cm, where bedrock was encountered. As was the case for test pit 2, the top 50 cm of test pit 3 belonged to the Iron Age (as well as more recent and modern times), the rest to the MSA (Fig 3). The MSA deposits are immediately below the Iron Age ones in this part of the site. (In other parts of Magubike, there are LSA occupations in between the Iron Age and MSA, but not here). Iron Age occupations are identified by the presence of ceramics, domesticated animal bones and, most importantly, by iron tools, and the remnants of iron smelting and blacksmithing activities (tuyeres, iron, slag, etc.) None of these are present in LSA or MSA levels. In test pit 3, there were numerous animal bones and large snail shells (mainly *Achatina* species) throughout the MSA levels [8–9]. In addition, six hominin teeth were recovered from the MSA deposits in test pit 3; all derived from the upper jaw. Four of these teeth came from between 130 and 140 cm (one central incisor, one canine and two premolars; the canine and one premolar were cemented together); the remaining two (one central and one lateral incisor) came from 150 to 160 cm below the surface.

**Fig 4:**
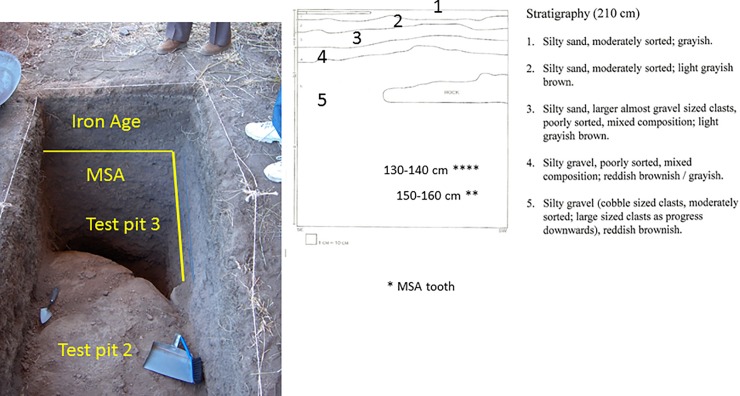
View of west wall of test pit 3 during excavations in 2006. The top 50 cm is composed of Iron Age and modern cultural deposits. Below that is Middle Stone Age, to bedrock at 210 cm below the surface.

The precise stratigraphic context of these finds is not clear (Fig 4). There are a series of layers in the top units that belong to the historic and Iron Age (units 1–4). The top two layers, comprising around 15 cm in depth, are both composed of moderately sorted silty sand. The top layer (unit 1) is greyish in colour, while unit 2 below is light greyish brown. The third layer is also light greyish brown, poorly sorted, and with a mixed composition of silty sand and larger, almost gravel-sized clasts. Unit 4 is reddish brownish/greyish and composed of silty gravel, it is poorly sorted, and has a mixed composition. The final layer (Unit 5, from around 50 cm to bedrock) is composed of silty gravel and cobble sized clasts; the latter increase in size with depth and represent decaying bedrock. This unit is reddish brownish (Biittner, personal observation). The stratigraphic sequence of test pit 12, excavated in 2012 and adjacent right to the west of test pit 3, has a similar stratigraphic sequence (Fig 4 in reference [81]). Here, six units were distinguished, which had an increase in clast size with depth and a change in colour from brown/grey to red was also observed. Note that it is test pit 12 that has supplied the bulk of the samples used for dating. The original description of the stratigraphic sequence of test pit 3 [4, 9] suggested that four of the teeth were recovered from a 10 cm deep spit (artificial layer of deposit) immediately below a 30 cm thick disturbed layer (i.e., one that contained a mixture of LSA and MSA artifacts), and that the other two teeth were separated from these by another 10 cm of deposit. Further excavations next to test pit 3 confirm that there are no LSA deposits in this part of the site and that the MSA levels begin between 30 and 50 cm below the modern surface. The fossil hominin teeth from 2006 come from well below this transitional layer.

**Fig 5:**
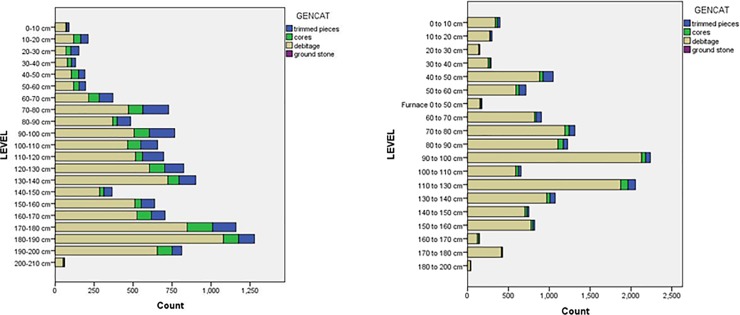
Number of stone artifacts by depth in test pits 3 (left) and 12 (right) at Magubike rockshelter.

**Fig 6:**
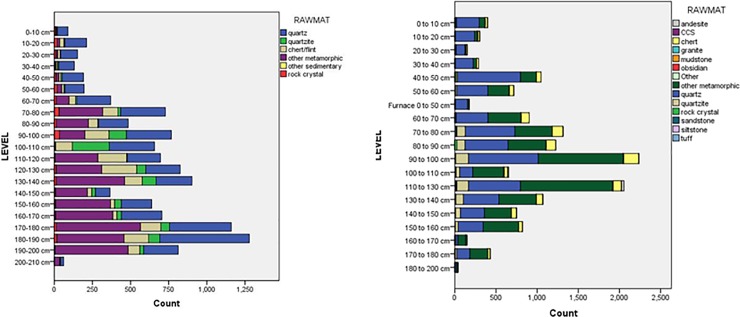
Lithic raw material frequency by depth in test pits 3 (left) and 12 (right).

A total of 11,417 stone artifacts were recovered from test pit 3 at Magubike. Of these, 1,749 (15.3%) are retouched tools, 1,295 (11.3%) are cores, 8,369 (73.3%) are debitage (including whole flakes and blades, chips and chunks), and 4 (0%) ground stone pieces (Fig 5). In terms of raw materials, 4,475 (or 39.2%) are quartz, 829 (7.3%) quartzite, 1,552 (13.6%) crypto-crystalline silica (chert/flint), 4,306 (37.7%) other metamorphic (i.e. not quartzite), 1 (0%) other sedimentary, and 254 (2.2%) are made of rock crystal (Fig 6). While in the Iron Age levels, quartz and chert are most common, metamorphic rocks dominate in the MSA levels.

**Fig 7:**
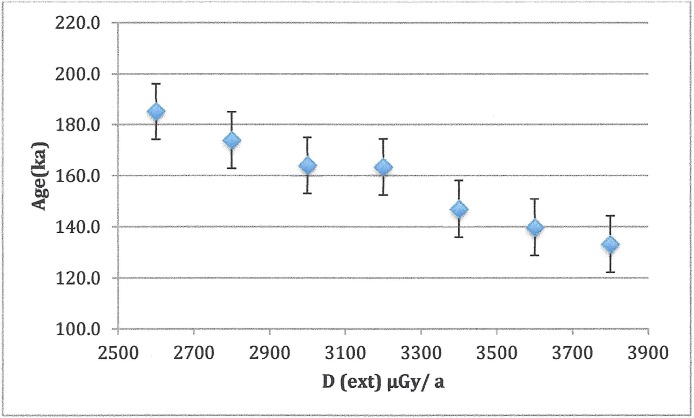
Effect of changing sedimentary dose assumptions on sample age. Error bars are ± 1σ.

Since the original field season, the main area of the site has been opened up further. A number of additional test pits were dug (numbering test pits 6 to 12) and samples were collected for various chronometric dating techniques. A plan view of the excavation areas is given in Fig 3, previously published in Werner and Willoughby [81]. Test pit 12 is immediately west and south of test pit 3 and was the source of numerous mollusc shells and mammal teeth which have been used for Electron Spin Resonance (ESR) dating. It measured 1 m by 1.35 m and extended to a level of 200 cm below the surface. A total of 14,738 stone artifacts were excavated from test pit 12. The archaeological sequence was identical to that in test pit 3, except that the change from the Iron Age to the MSA occurred at a depth of 40 cm below the surface (rather than at 50 cm as in test pit 3). The only difference was in the west wall of test pit 12, where the remains of an iron furnace extended to a depth of 50 cm (see “Furnace 0 to 50 cm” in the level categories for Figs 6 and 7). Of the 14,739 stone artifacts, 799 (5.4%) are retouched tools, 1,574 (3.9%) cores, 13,331 (90.5%) debitage and 4 (0%) are ground stone pieces (Fig 5). There are a smaller percentage of retouched tools in test pit 12, but this may reflect a better understanding of what counts as a “tool” at this site. Most of the retouched tools come from top levels; MSA artifacts tend to not be retouched. In terms of lithic raw materials, the two test pits are quite similar. But by the time test pit 12 was excavated, we had developed a more complex classification for lithic raw materials based on Biittner’s work [6]. Quartz makes up 44% of the lithic raw materials (n = 6,484 pieces) while “other metamorphic” is the next most common at 40.7% (n = 6,002) Chert comprises 7.6% (n = 1,119) and quartzite 5.6% (n = 826) (Fig 6).

In conclusion, test pits 12 and 3 are immediately adjacent to one another and contain a similar archaeological sequence. The types of lithic artifacts and raw material frequencies are remarkably similar. Test pit 12 was excavated with a view to recovering in situ samples of shells and mammal teeth for ESR dating. We are confident that the dates from test pit 12 reflect the age of test pit 3, and therefore, for the hominin teeth described here.

## Dating of Magubike shells, ostrich eggshell beads and mammal teeth

ESR dating relies on the accumulation of radioactive damage over time [10], for which tooth enamel is the preferred study material. Signal stability in tooth enamel is extremely high [11], and because tooth enamel is highly mineralized, it is often preserved in sites. At Magubike approximately a dozen teeth or tooth fragments of medium to large mammals, primarily equids, were recovered from various levels in different test pits. The list of samples is presented in Table 1.

**Table 1:** Tooth samples from Magubike rockshelter.

Catalogue	Sample	Test Pit	Depth (cm)
2012MAG04	PT86	8	110–120
2012MAG07	PT82	8	130–140
2012MAG10	PT83	8	130–140
2012MAG01	PT85	9	30–40
2013MAG45a	PT90	9	50–60
2013MAG50a	ET25	12	80–90
2013MAG51a	PT96	12	130–140
2013MAG53a	PT98	12	170–180

**Fig 8:**
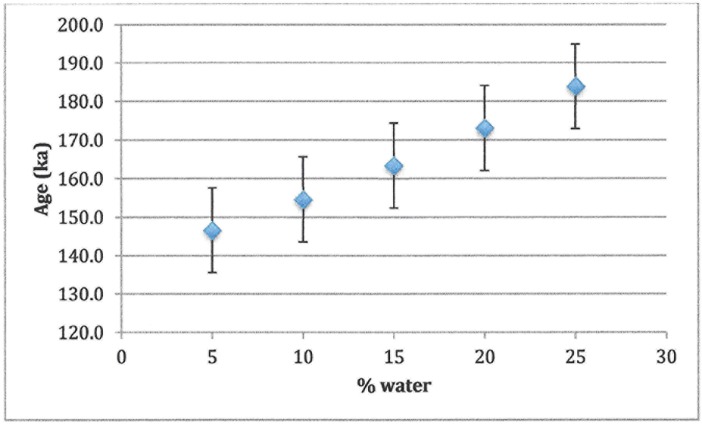
Effect of varying assumed water concentration on sample age. Error bars are ± 1σ.

Samples were prepared, measured and analysed by standard methods [12]. Dentine was removed by mechanical drilling with a hand-held Dremel drill. Once all dentine had been cleaned off, the sample thickness was measured by a micrometer and an additional 20 μm of enamel was removed from each side, in order to eliminate the effects of alpha radiation from the dentine. A Cs-137 source with a dose rate of ~2.5 Gy/min was used for the artificial irradiation. At minimum, 10 doses (maximum dose 5x-10x AD) were used to determine the accumulated dose (AD) or total radiation received by the sample. Samples that had not been reirradiated were run along with the ramped aliquots to check for spectrometer drift. ESR results were acquired on a JEOL RE1X spectrometer at a power of 2 mW and modulation amplitude of 0.1 mT. Plotting ESR intensity against dose gave the AD. Typical spectra and growth curves are shown in Fig 7. Environmental and internal dose rates were calculated using neutron activation analysis (NAA) to determine concentrations of radioisotopes. Since anything in the environment within 30 cm contributes to the dose rate, in addition to the sediment in the level where the tooth was found, sediment from levels above and below were averaged in. In calculating ages, cosmic radiation was neglected due to the extent of cover over the rockshelter and the small angle of opening [13]. Sedimentary water was assumed to be the modern value, 10% ± 5%. Sediment dose rates were derived from sediment collected within 30 cm of the tooth, as well as samples of the rock wall. However, testing the sedimentary assumptions showed that the external dose rate could vary by as much as 20% in either direction without changing the calculated ages outside ±2σ (Fig 7). Similarly, changing water concentration did not change ages significantly (Fig 8).

A complication when dating tooth enamel is that teeth are semi-open systems. Dentine and enamel take up uranium during burial. The [U] in tooth enamel and dentine is shown in Table 2. Traditionally, we report three possible ages depending on assumptions about the rate of uptake. Early uptake (EU) assumes that the modern concentration of uranium was achieved shortly after burial, yielding the youngest age. Linear uptake (LU) assumes that uranium has accumulated relatively steadily during burial, and recent uptake (RU) assumes that, due to some change in the environment or the tooth, most uranium accumulated relatively recently before excavation, yielding the oldest ages. Complex uptake models are also possible, but these three cover most cases, with RU being generally rare. In this study, RU ages have not been considered because they are clearly not probable. When comparison dating methods have been used [14], best agreement has come with LU ages. This is not surprising in a site where water, the source of U, has been able to percolate through the sediment. In the last few decades, coupled U-series/ESR dating has made it possible to refine the uptake model. Samples have been submitted for this additional technique but to date none have yielded results.

**Table 2:** Uranium content for tooth enamel and dentine. Uncertainty for all values is 0.02 ppm.

Sample	[U]en (ppm)	[U]den1 (ppm)	[U]den2 (ppm)
PT86en1	0.15	16.14	16.14
PT86en2	1.38	15.26	11.18
PT86en3	0.75	11.18	15.26
PT82en4	0.56	9.10	9.10
PT82en2	0.23	6.30	9.10
PT82en6	0.55	5.51	6.58
PT82en5	0.84	6.14	6.14
PT85en1	0.13	7.58	6.46
PT85en2	0.15	6.46	7.17
PT85en3	0.32	6.76	6.46
PT90en1	0.65	14.41	9.57
PT90en2	0.56	13.07	9.57
PT90en3	0.66	9.57	13.74
ET25en2	0.49	10.66	33.17
ET25en1	0.37	10.78	45.67
PT96en1	0.77	29.58	29.58
PT96en3	2.35 (est)	23.52	23.52
PT98en1	0.85	20.04	13.90
PT98en2	1.29	13.90	19.75
PT98en3	0.50	19.46	13.90

**Fig 9:**
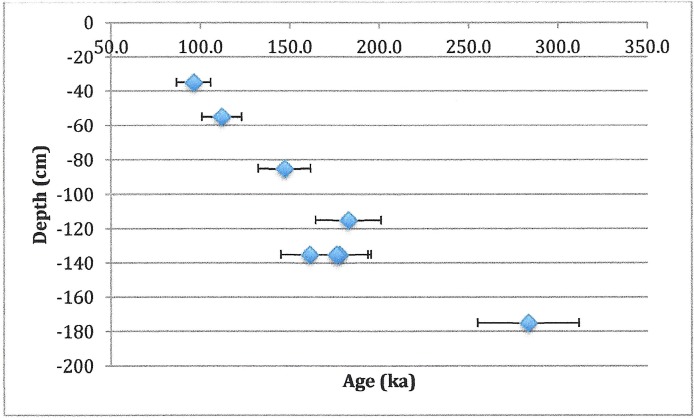
Age as a function of sample depth. Where multiple subsamples were measured, ages are average of all subsamples.

Results are presented in Table 3 and the LU ages are plotted in Fig 9. Additional information is provided in the S1 File.

Overall, samples show stratigraphic consistency. The data from Test Pit 12, 10 to 65 cm south of where the hominin teeth were found, suggests a minimal age for the 130–140 cm level of 160–180 ka, and an age for the 170–180 cm level of 280 ka. The latter age is from a single tooth fragment and clearly additional dates would be useful to rule out reworking.

**Table 3:** Summary ages for tooth samples. D(ext) is the external dose rate. EU D(int) is the internal dose rate assuming an early uptake model. EU is the age in ka assuming an early uptake model. LU D(int) is the internal dose rate assuming a linear uptake model. LU is the age in ka assuming a linear uptake model. Does units are in micrograys per year. Sedimentary water concentration: 10%. Alpha-efficiency: 0.13.

Sample		D (ext) μGy/a	AD (Gy)	±Gy/a	EU (ka)	LU D (int) μGy/a	LU (ka)
PT86en1		3260	815.0	1520	170.5	702	205.7
	±	261	32.0	43	12.9	17	17.6
PT86en2		3260	763.6	1299	166.7	605	197.6
	±	261	25.0	41	12.5	17	16.7
PT86en3		3260	711.4	1183	160.1	543	187.1
	±	261	31.2	30	13.0	13	16.9
PT86 (avg)		3260			169.0		196.8
	±	261			12.7		17.0
PT87en1		3260	799.8	1401	167.3	642	199.9
	±	261	98.1	41	24.3	18	30.3
PT82en1		4231	678.2	538	142.2	242	151.7
	±	306	48.0	15	13.6	9	14.9
PT82en4		4231	735.0	639	155.2	286	167.7
	±	298	28.0	23	11.5	9	13.1
PT82en6		4231	745.0	400	160.9	177	169.0
	±	298	50.0	25	15.1	12	16.2
PT82en5		4231	670.0	585	143.0	269	153.7
	±	298	23.0	23	10.4	10	11.8
PT82 (avg)		4231			150.3		160.5
	±	298			12.3		13.1
PT83en1		4099	830.0	2850	120.3	1221	152.7
	±	298	38.7	57	7.8	27	11.1
Sample		D (ext) μGy/a	AD (Gy)	EU D (int) μGy/a	EU (ka)	LU D (int) μGy/a	LU (ka)
PT85en1		3211	283.0	429	77.7	195	83.1
	±	202	15.0	9	6.0	4	6.6
PT85en2		3211	267.0	381	74.3	174	78.8
	±	202	10.0	8	5.0	3	5.6
PT85en3		3211	299.8	431	82.3	196	88.0
	±	202	10.0	12	5.3	4	6.0
PT85 (avg)		3211			78.0		83.3
	±	202			4.1		6.0
PT90en1		4890	555.0	737	98.6	333	106.3
	±	306	19.0	18	6.4	8	7.2
PT90en2		4890	547.3	679	98.3	307	105.2
	±	306	28.1	17	7.4	7	8.2
PT90en3		4890	549.8	622	98.0	327	105.4
	±	306	17.9	18	6.3	8	7.1
PT90 (avg)		4890			98.3		105.6
	±	306			6.5		7.5
PT91en1		4796	744.6	1056	127.2	482	141.1
		354	21.8	32	8.6	14	10.3
PT91en2		4796	616.4	733	113.6	285	121.3
		354	26.0	21	8.9	9	9.9
PT91 (avg)		4796			120.8		131.1
		354			8.8		10.1
ET25en2		4959	915.0	1245	138.0	616	155.0
	±	328	45.4	45	7.1	20	7.8
ET25en1		4959	696.3	1382	104.5	638	117.6
	±	328	45.4	34	8.6	22	10.1
ET25 (avg)		4959			121.3		135.8
	±	328			7.8		9.0
Sample		D (ext) μGy/a	AD (Gy)	EU D (int) μGy/a	EU (ka)	LU D (int) μGy/a	LU (ka)
PT96en1		3878	798.8	2118	133.2	990	164.1
	±	203	54.0	90	10.7	35	13.2
PT96en2		3878	1018.0	2176	168.2	1016	208.1
	±	203	60.0	89	14.1	41	18.3
PT96 (avg)		3878			150.7		186.1
	±	203			12.4		15.7
PT98en1		3391	1121.7	1513	229.5	705	274.7
	±	213	35.3	35	12.8	18	16.8
PT98en2		3391	781.1	1524	159.0	703	190.8
	±	213	37.4	41	10.6	18	13.6
PT98en3		3391	966.5	1350	203.9	725	240.8
	±	213	30.0	32	11.4	14	14.9
PT98(avg)		3391			194.1		235.4
	±	213			11.7		15.1

Molluscs are another potential material for dating [10]. Multiple samples of *Achatina* sp. have been found at Magubike. Some were prepared and analysed by standard methods [132]. However, in instances where teeth and shells were from the same level in the same test pit, for example in test pit 12, it was found that the ages of the shells were considerably older, by as much as 100 ka. As *Achatina* is a burrowing species, it should yield ages less than or equal to tooth ages for a site. This is particularly true for these samples. The snails in the MSA levels tend to be whole, suggesting that they may have lived in the shelter and burrowed into the MSA deposits to estivate. Close examination indicates an impurity in the ESR spectrum that distorts the results. More study is underway to see if these ages in can be corrected. In the meantime, shell ages from Magubike, such as those published in Werner and Willoughby (2017), have to be discarded.

Further evidence that the shell ages are incorrect is shown by comparison of ESR and 14C ages. Initially, the Isotrace Radiocarbon laboratory at the University of Toronto dated two *Achatina* snail shells. One from the Iron Age / historic levels from 20 to 30 cm produced an uncalibrated date of 2,990 ± 60 BP (TO-13422); this produces a 68% confidence interval of 1,315 to 1,125 calibrated years BC. Another, from 130 to 140 cm, the same level as four of the hominin teeth, produced an uncalibrated date of 41,790 ± 690 BP. Three shells from the 2012 excavations, which were dated by radiocarbon, were also dated by ESR (Table 4). All came from test pit 12, which is immediately adjacent to test pit 3 from 2006. One shell, from the Iron Age levels, 20 to 30 cm below the surface, produced an uncalibrated date of 4,477 ± 32 years BP (OxA-27438). Another shell, from close to the top of the MSA deposits (60 to 70 cm below the surface), gave a date of 49,200 ± 900 BP (between 49,403 and 45,600 cal BC) (OxA-27439). The third shell, from 90 to 100 cm below the surface gave a similar date: 47,550 ± 700 BP (or between 47,218 and 44,291 cal BC) (OxA-27440). These dates are consistent with the original *Achatina* date from Isotrace. In contrast, and consistent with the original Isotrace results, the ESR ages are much older. *Achatina* shells seem to give radiocarbon dates that are similar to the ESR dates when they come from the recent sequence but appear to be much younger than expected when they come from the MSA deposits. One could hypothesize that the radiocarbon ages are minima and the ESR ages maxima, but the difference between them is too large to make this a realistic proposition. The conservative assumption, then, is that the radiocarbon dates provide at least a minimum age for these deposits, and that the MSA occupation in the main part of the Magubike rockshelter is at least 40,000 to 50,000 years old.

**Table 4:** Comparison of ESR and ^14^C ages for *Achatina* shells.

Catalogue	Sample	Depth	C-14 Sample	ESR Age (ka)	C-14 Age (ka)
2015MAG02	CM69	20–30	OxA-27438	6.2 ± 0.4	4.47 ± 0.03
2015MAG01	CM67	60–70	OxA-27439	202 ± 20	49.2 ± 0.9
2015MAG03	CM68	90–100	OxA-27440	183 ± 18	47.6± 0.7

Along with the snail shells, a number of ostrich eggshell beads (OES) and bead fragments from test pit 12 were submitted for radiocarbon dating [15]. Three of these came from MSA levels while two came from the Iron Age / historic levels.

One OES bead disc or preform came from 80 to 90 cm below the surface in test pit 12. It produced a date of 31,810 ± 180 BP (with a 72% probability of an age between 34,798 to 34,239 cal BC, and a 23.4% probability the age ranges from 34,015 to 33,585 BC) (OxA-27627). A second preform from 70 to 80cm below the surface in test pit 12 gave a date of 47,750 ± 750 BP (47,405 to 44,418 cal BC) (OxA-27626). The third, a complete bead, came from between 90 and 100 cm below the surface in test pit 11, east of test pit 12 and south of test pits 2 and 3 from 2006. This had a radiocarbon date of greater than 50,100 BC (OxA-27628) [16].

Given the stratigraphic depth at which the human teeth were found in test pit 3, they are likely to be at least 45,000 years old, and perhaps much older. In the future, it may be possible to directly date the teeth by laser ablation using ESR and U-series. In addition to the radiocarbon and ESR dating, James Feathers of the University of Washington is also attempting to date sediments from test pit 12 using optically stimulated luminescence (OSL). However, no final dates are available yet.

## Materials and methods

**Fig 10:**
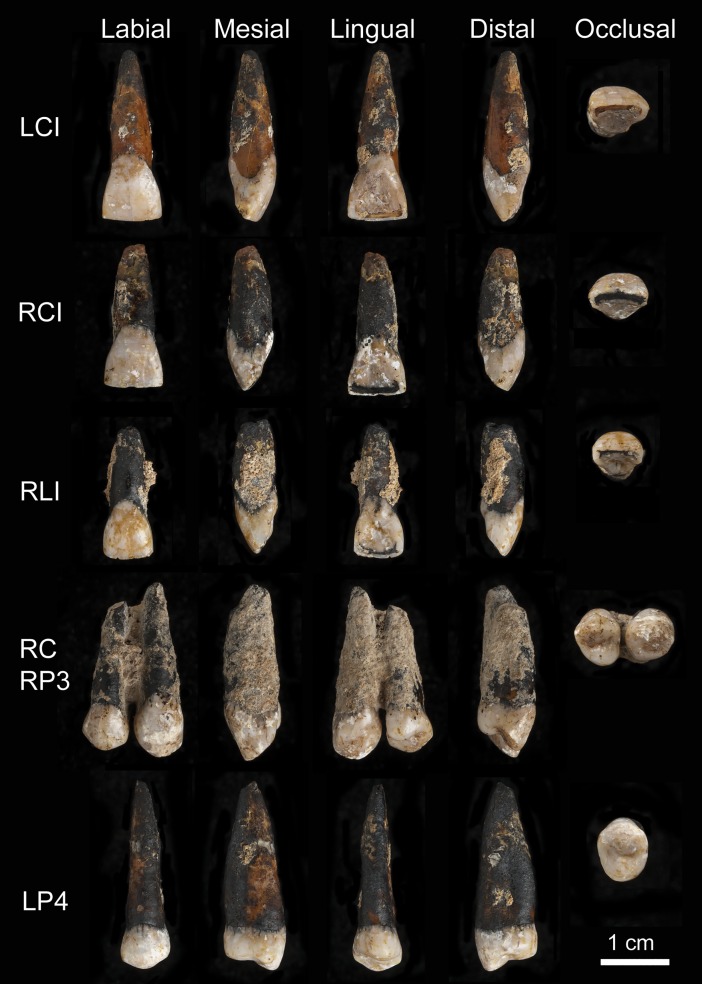
Photos of the labial, mesial, lingual, distal and occlusal surfaces of the Magubike teeth: Upper left central (LCI), right central (RCI) and right lateral (RLI) incisors, right canine (RC) and right third premolar (RP3) and left fourth premolar (LP4). Scale 1cm.

The material available for study consists of six permanent, fully developed upper teeth. They are: left and right central incisors, right lateral incisor, right canine, right third premolar and left fourth premolar (Fig 10). They were all isolated, without any associated bone. However, the right canine and right third premolar are held together by matrix and by a fragment of alveolar bone at a point two-thirds of the way up the root. Aspects of the morphology, metrics, wear, and pathology of the teeth suggest that they come from the one individual.

Four samples were prepared for making morphological comparisons:

1. African mid/late Pleistocene, based on published descriptions, casts, and original specimens held by the Human Origins Group in the Department of Earth Sciences at the Natural History Museum (NHM), London. Sites in this sample fall into two principal groups: a) Middle Pleistocene pre-modern hominins assigned to *Homo heidelbergensis* or archaic *Homo sapiens* (Thomas Quarries III, Salé, Rabat, Jebel Irhoud, Broken Hill, Eyasi); b) mid/late Pleistocene *Homo sapiens* (Guomde, Sea Harvest Cave, Kabua 1, Die Kelders Cave, Equus Cave, Nazlet Khater, Wadi Kubbaniya and the Aterian sites of Mugharet el ‘Aliya, Dar es Soltan, and Smuggler’s Cave). The morphological traits that can be determined vary with the level of wear, and for some sites, little useful information can be gleaned. Due to the scarcity of relatively unworn sub-Saharan material, the sample has a North African bias. References for sites included in this sample are identified in Table 5. The data for number of premolar roots for Ndutu, Jebel Irhoud, Ngaloba LH18 and Bodo is from Schwartz and Tattersall [112]. ˟: Crown measurements are included in groups listed in Table 6. Dates are taken from Klein [113] and other cited references.
2. African Holocene material held at the NHM, London, for making both metrical and morphological comparisons:
  - a Kenyan early Holocene sample consisting of specimens from the sites of Elmenteita, Naivasha, Naishi and Gamble's Cave [17];
  - specimens from the site of Gwisho (Lochinvar) in Zambia, dated at 5–4 ka [18].
3. Recent East/South African (last 1000 years) utilising sub-Saharan material held at the NHM, London, for traits for which there are little relevant published data, principally relating to premolars.

**Table 5:** References used for comparative material. Headings relate to groupings of sites used in Table 6.

Specimen	Reference
***Homo erectus***	
‘*Sinanthropus*’ (China ~770 ka)	Weidenreich 1937 [23]
KNM-ER1808˟ (~1.8–1.7 Ma)	Brown and Walker 1993 [82]
KNM-ER3733˟ (~1.8–1.7 Ma)	Leakey and Walker 1985 [83]
SK27˟ (~1.8–1.5 Ma)	Clarke 1977 [84]
KNM-ER803˟ (~1.6–1.4 Ma)	Brown and Walker 1993 [82]
Nariokotome˟ KNM-WT15000 (~1.5 Ma)	Brown and Walker 1993 [82]
OH 59/29˟ (~1.15–0.8 Ma)	Rightmire 1980 [85]
***Homo heidelbergensis /* archaic *Homo sapiens***	
Tighennif˟ (Algeria ~780 ka)	Arambourg and Hoffstetter 1963 [87]
Thomas Quarries III*˟ (Morocco ~600–400 ka)	Ennouchi 1976 [88]; Rightmire 1990 [86]
Salé*˟ (Morocco ~400 ka)	Jaeger 1975 [89] ; Day 1986 [91]; cast
Jebel Irhoud*˟ (Morocco ~350–300 ka)	Hublin et al. 2017 [2] Richter et al. 2017 [125]
Rabat*˟ (Morocco ~200–130 ka)	Day 1967 [90]; Thoma and Vallois 1977 [42]
Broken Hill*˟ (Zambia ~300–200 ka)	Carter 1928 [92]; Day 1986 [91]; original specimens
Lainyamok˟ (Kenya ~392–330 ka)	Shipman et al. 1983 [93]
Wadi Dagadlé˟ (Djibouti ~400–250 ka)	De Bonis et al. 1984 [94]
Eyasi*˟ (Tanzania ~400–200 ka)	Weinert et al. 1939 [95]
**Aterian**	
Mugharet el ‘Aliya*˟ (Morocco ~128–40 ka)	Şenyürek 1940 [96]; Briggs 1967 [53]
Dar es Soltan*˟ (Morocco ~128–40 ka)	Hublin et al. 2012 [97]
Smuggler's Cave (Grotte des Contrebandiers)*˟ (Morocco ~128–40 ka)	Hublin et al. 2012 [97]
**Sub-Saharan Middle Stone Age *Homo sapiens***	
Guomde* (Kenya ~300–100 ka)	Photographs taken by G Bräuer, F Spoor; cast
Herto˟ (Ethiopia ~160–154 ka)	White et al. 2003 [98]
Omo Kibish 1˟ (Ethiopia ~198–104 ka)	Leakey et al. 1969 [99]
Sea Harvest Cave*˟ (South Africa ~128–40 ka)	Grine and Klein 1993 [120]
Kabua 1*˟ (Kenya)	Whitworth 1966 [117]; original specimen
Die Kelders Cave*˟ (South Africa ~75–65 ka)	Avery et al 1997 [100]; Grine 1998 [101], 2000 [74]
Equus Cave*˟ (South Africa ~75–27 ka)	Grine and Klein 1985 [102]
**North African later late Pleistocene / early Holocene**	
Nazlet Khater* (Egypt MSA ~37.5 ka)	Thoma 1984 [103]; Pinhasi 1998 [104]
Jebel Sahaba˟ (N Sudan ~14–12 ka)	Anderson 1968 [105]
Wadi Halfa˟ (N Sudan ~11–8 ka)	Greene et al. 1967 [106]
Wadi Kubbaniya* (Egypt ~25–20 ka)	Wendorf and Schild 1986 [107]
N African Epipal/Meso˟ (Taforalt and Afalou)	Bermúdez de Castro 1991 [108]
**Sub-Saharan Holocene**	
Matjes River Proto Bushman (South Africa ~9.7 ka)	Keith 1933 [109]; Sealy et al. 2006 [110]; cast ("proto-Bushman)
Kenya early Holocene (Elmenteita; Naivasha; Naishi; Gamble's Cave)	Measured by Tim Compton
Gwisho (Zambia ~5–4 ka)	Measured by Tim Compton
Mapungubwe (South African Iron Age)	Galloway 1937 [56]
**Other**	
Mugharet es-Skhūl (Israel ~120–90 ka)	McCown and Keith 1939 [118]
**Recent sub-Saharan (measurements)**	
Teso (Kenya)	Barnes 1969 [111]
San Bushmen	Drennan 1929 [79]
Bantu (South African)	Middleton Shaw 1931 [57] (crown heights and root lengths); Jacobson 1982 [54] (crown dimensions)

*: Sites included in mid/late Pleistocene morphological sample.

**Table 6:** Comparative crown measurements (in mm).

Site / Sample	Upper I1 ‘n’	Upper I1 Area	Upper I1 Index	Upper I2 ‘n’	Upper I2 Area	Upper I2 Index	Upper C ‘n’	Upper C Area	Upper C Index	Upper P3 ‘n’	Upper P3 Area	Upper P3 Index	Upper P4 ‘n’	Upper P4 Area	Upper P4 Index
**African non-recent**															
*Homo erectus* Ẋ	3	94	75	3	66	102	3	96	98	4	113	144	2	98	145
*Homo erectus* Range		77–110	71–80		62–72	99–105		79–110	91–106		101–129	137–162		98	142–149
*H*. *heidelbergensis* / archaic *H*. *sapiens* Ẋ				2	72	97	7	92	110	5	92	138	6	93	142
*H*. *heidelbergensis* / archaic *H*. *sapiens* Range					68–76	88–106		81–110	100–126		82–105	132–147		79–105	135–156
Aterian (128–40 ka) Ẋ							1	89	133	2	82	126	2	103	129
Aterian (128–40 ka) Range											79–84	119–134		102–103	118–140
Magubike Ẋ	2	75	80	1	58	87	1	71	117	1	70	128	1	69	126
Sub-Saharan Middle Stone Age Ẋ	2	61	72	1	37	102	4	73	106	2	71	145	7	72	132
Sub-Saharan Middle Stone Age Range		60–61	71–73					58–89	91–119		60–81	124–167		54–81	122–150
N African later Late Pleistocene / early Holocene Ẋ	17	62	71	33	51	94	34	71	113	36	75	133	44	72	140
Kenya (Early Holocene) Ẋ	2	67	88	4	49	97	5	64	111	10	72	131	11	66	140
Kenya (Early Holocene) SD		5	1		8	11		5	9		8	10		8	7
Kenya (Early Holocene) Range		64–70	88–89		43–60	87–111		59–71	101–126		64–87	110–145		56–80	131–154
Gwisho (Zambia, 5–4 ka) Ẋ	8	60	82	6	42	94	6	59	105	7	62	132	6	65	136
Gwisho (Zambia, 5–4 ka) SD		9	9		5	6		11	3		8	8		7	8
Gwisho (Zambia, 5–4 ka) Range		49–71	73–97		35–48	86–100		47–76	100–108		52–75	120–143		55–74	127–148
Mapungubwe (SA, Iron Age) Ẋ	4	69	88	4	58	92	3	82	110	3	86	134	4	85	133
**Recent Sub-Saharan**															
Teso (Kenya) Ẋ	27	53	82	28	46	91	15	67	106	14	75	131	10	72.6	132.9
Teso (Kenya) Range											56–95	124–147		58–80	119–147
San Bushman Ẋ	25	54	78	24	40	90	26	59	104	25	58	126	26	55	131
antu (SA) Ẋ	191	63	83	266	46	95	363	65	109	389	68	132	386	64	140
Bantu (SA) Range		48–86	70–110		24–85	57–124		45–91	92–139		44–90	108–150		45–86	121–163

In addition, principally for traits in the anterior teeth, Bailey's 2006 [16] sample of early modern Afro-Asians, mostly containing teeth from Skhūl and Qafzeh, but also from Die Kelders Cave, Equus Cave and Sea Harvest Cave, was employed and, in addition, published samples of recent Bantu and recent San.

Published morphological data predominantly relate to anterior teeth, of the teeth being studied, and not to premolars. Consequently, whilst the mid/late Pleistocene and Kenyan early Holocene samples are used for comparison to all the teeth, the early modern Afro-Asian and recent Bantu and San samples are used for comparison to the anterior teeth, and the recent East/South African sample is used for comparison to the premolars.

Headings in Table 5 relate to the groupings of non-recent sites used for making metrical comparisons in Table 6. Sites included in each group are identified by ‘˟’. Comparisons are also made to metrical data published for recent African groups: Teso, Bantu, and San Bushmen. Measurements for comparative material used in Tables 6, 7 and 8 are taken from published sources, as indicated in Table 5, unless otherwise stated.

**Table 7:** Comparative measurements for crown height (in mm).

Site / Sample	Upper Central Incisor N	Upper Lateral Incisor N	Upper Canine N	Upper Third Premolar N	Upper Fourth Premolar N
Magubike	>11.3*	>10.0	10.7	7.9	6.8
SK27 Ẋ		1 14.0†	1 16.3		
Nariokotome	1 12.95	1 13.3*	1 11.9	1 9.4	1 8.05
'*Sinanthropus*' Ẋ	1 13.3	2 11.65	2 13.9	1 9.7	2 8.25
'*Sinanthropus*' Range		11.4–11.9	13.6–14.2		8.2–8.3
Rabat		1 >10.0	1 12.5	1 ≈ 10.0	1 8.5
Mugharet el Aliya			1 12.9	1 9.0	
Sea Harvest					1 8.5
Equus Cave		1 10.2			
Kenya Early Holocene Ẋ	2 10.6	4 9.8	4 10.4	8 8.3	6 7.9
Kenya Early Holocene Range	10.0–11.1	8.5–10.8	9.9–10.8	7.9–9.1	7.3–8.5
Gwisho Ẋ	2 11.5	1 9.6	1 11.2	2 7.6	1 >7.0
Gwisho Range				7.0–8.1	
**Recent**					
San Bushmen Ẋ	19 9.3	18 7.9	20 7.9	17 6.6	15 6.3
Bantu Ẋ (range)	65 10.5	66 9.4	66 9.9	62 7.9	87 7.7
Bantu Range	9.0–12.5	8.0–11.0	8.5–12.0	7.0–8.5	6.5–9.0

*: Highest of two antimeres shown. Sea Harvest UP4 measured from scaled photograph.

†: Measured lingually.

**Table 8:** Comparative measurements for root length (in mm).

Site / Sample	Upper Central Incisor		Upper Lateral Incisor		Upper Canine		Upper Third Premolar		Upper Fourth Premolar	
	N	Length	N	Length	N	Length	N	Length	N	Length
Magubike—Actual		16.5 (left)		>12.1		>18.1		>17.0		21.6
Magubik—Estimate*				16.5		19.5		20.0		
Nariokotome	1	21.15	1	>18.2						
*'Sinanthropus’ Ẋ*	4	17.1	1	19.0	4	22.6	2	20.2	3	15.2
*'Sinanthropus’* Range		11.5–0.7				21.8–23.2		20.0–20.4		13.3–16.2
Skhūl Ẋ	3	17.3	3	18.2	4	18.6	2	15.5	3	14.7
Skhūl Range		15.5–20.0		17.8–18.5		15.0–22.8		11.6–19.3		13.3–17.5
Rabat					1	>17.5	1	≈ 18.5	1	19.0
Sea Harvest									1	>19.1
Klasies River Main			1	13.5						
Equus Cave	1	14.0	1	12.5	1	18.7				
Matjes River	1	17.5								
Gwisho Ẋ	6	13.5	4	13.8	2	15.4	2	14.0	3	14.6
Gwisho Range		11.5–16.9		11.4–18.0		14.6–16.0		12.7–15.2		12.2–16.9
Mapungubwe Ẋ	2	15.5	2	12.3	2	18.3	1	15.0	2	16.0
Mapungubwe Range		14.0–17.0		11.5–13.0		17.5–19.0				15.0–17.0
**Recent**										
Teso Ẋ	19	13.8	20	13.7	15	17.9	15	15.0	10	16.4
Teso Range		12.0–16.3		12.0–17.3		14.8–24.2		13.6–18.0		13.2–18.3
San Bushmen Ẋ	19	12.5	18	12.5	20	16.3	17	14.5	15	14.8
San Bushmen Range		10.0–15.5		11.0–16.0		13.3–19.5		12.0–18.0		13.0–18.3
Bantu Ẋ	66	13.4	66	13.6	66	17.3	62	13.9	87	14.6
Bantu Range		11–18		10–16		13–24		10–19		13–19.5

*: Estimated lengths of Magubike roots calculated by extending the buccal and lingual surfaces and assuming a rounded apex.

The morphological traits studied are described in Table 9. Most of the morphological traits studied are in the ASU (Arizona State University) dental anthropology system (ASUDAS), and the ASU scoring system is followed using their dental reference plaques [19–20]. Where both left and right antimeres are present, the highest expression of a trait encountered is the one used (according to [19]).

**Table 9:** Descriptions of non-metric traits used in this study.

*Bilateral winging* I^1^: Both central incisors are rotated mesiolingually, giving a v-shaped appearance
*Crown shape* (viewed from buccal aspect) I^1^: Scored as square (type 1), tapering (type 2) or ovoid (type 3) using Leon Williams' classification (Williams [121]).
*Shovelling* I^1^, I^2^, C^1^: The presence of mesial and distal lingual marginal ridges. Degree scored as grades 1–7.
Note on measurement of degree of shovelling:—Jacobson [54] and Barnes [111] record shovelling as trace, semi and full. In using this method, Carbonell [119] defined semi-shovel as being when the depth of the lingual fossa between the mesial and distal margins was 1 mm. Trace shovel had a depth less than this and full shovel had a depth greater. The depth of the lingual fossa on grade 2 of the ASUDAS UI1 shovelling dental plaque is 1 mm and grade 2 is therefore equivalent to semi-shovel.
*Double shovelling* I^1^, I^2^, C^1^, P^3^, P^4^: The presence of mesial and distal buccal (labial) marginal ridges. Degree scored as grades 1–6. Note: Defined in the ASUDAS system as referring to anterior teeth and third premolars but here also applied to fourth premolars.
*Occlusal labial convexity* (viewed from occlusal aspect) I^1^: The extent to which the labial surface of the incisor is curved. Degree scored as grades 0–4.
*Interruption groove* I^1^, I^2^: A groove that crosses the cingulum on the lingual tooth surface, mesial, distal or medial, often reaching the root.
*Tuberculum dentale* (including finger-like projections) I^1^, I^2^, C^1^: Emanating from the cingular region of the lingual surface. Can range from the form of finger-like projections (grades 1–4) to a cusp or tubercle, with or without a free apex (grades 5- to 6).
*Crown shape*, *peg/reduced* (viewed from buccal aspect) I^2^: May be normal, or distally reduced, reduced in size, peg-shaped or have a distal indentation (Hillson [51]). (This is scored as reduced in size or peg-shaped in the ASUDAS system.)
*Cingulum pit* I^2^: A pit occurring in the centre of the lingual cingulum. May be small or large and hollowed out (*dens invaginatus*). Grades 1–2.
*Occlusal shape round* (viewed from occlusal aspect) C^1^: The occlusal shape may be swollen (especially lingually) and rounded (premolariform), or triangular / diamond shaped, or mesiodistally elongated.
*Occlusal mesiobuccal bulge* (viewed from occlusal aspect) C^1^: There is commonly a mesiobuccal bulge in the occlusal outline.
*Buccal basal prominence* (viewed in vertical position from mesial aspect) C^1^, P^3^, P^4^: The buccal surface extends significantly beyond the line of the cervix.
*Lingual longitudinal ridge (tubercle extension)* C^1^: Extends from the tuberculum dentale to the occlusal edge, may be placed centrally or mesially, and can be multiple. It can be replaced by a deep central groove (not scored).
*Canine mesial ridge* C^1^: Mesiolingual ridge is larger than the distolingual ridge and is attached to the lingual tubercle such that it runs without interruption to the distal side of the tooth.
*Distal accessory ridge* C^1^: A ridge occurring in the distolingual fossa between the tooth apex and the distal marginal ridge. Size scored as grades 1–5.
*Root buccal vertical curvature* (viewed from mesial aspect) I^2^, C^1^: There is distinct convexity of the buccal side of the root, in particular towards the apex, such that the apex is placed lingually to the vertical axis of the tooth. Determined from root sockets for sub-Saharan East and South African sample.
*Accessory marginal cusp* P^3^, P^4^: The sagittal sulcus is strongly bifurcated at its mesial and/or distal end resulting in a bulge or free-standing accessory tubercle on the marginal ridge.
*Buccal accessory occlusal ridge* P^3^, P^4^: The presence of mesial or distal accessory ridges on the buccal cusp running near parallel to the essential crest.
*Buccal essential crest divided* P3, P4: The buccal essential crest may bifurcate as it approaches the sagittal sulcus.
*Sagittal sulcus interrupted (transverse crest)* P^3^, P^4^: The sagittal sulcus (mesiodistal occlusal developmental groove) may be continuous, or be interrupted by one or, very rarely, more enamel ridges connecting the buccal and lingual cusps. This may be medially, mesially or distally placed. (Kraus and Furr [114]; Ludwig [115]).
*Number of roots* on P^3^, P^4^: There may be one root, two roots, buccal and lingual, or three, resulting from bifurcation of the buccal root.

The definitions used for measuring mesiodistal and buccolingual crown diameters, crown height and root length are those of Moorrees [21], and those for root robusticity are as defined in Compton and Stringer [22], based on Weidenreich's method [23]. Cervical measurements are those defined by Hillson *et al*. [24]. No adjustments for wear were made to the mesiodistal crown dimensions of the Magubike teeth, it not being considered that the interproximal wear was sufficient to warrant this. Where both antimeres were measured, the measurements are averaged unless otherwise stated. Estimated root lengths were determined by extending the buccal and lingual surfaces of the root on a scaled photograph and assuming a narrow rounded tip similar to those on the two teeth with complete roots. The method of Smith [25] is employed to describe the level of wear on the teeth.

All the observations made on the Magubike teeth were repeated after an interval of a month. On other material, outlier measurements and observations of low frequency traits were repeated.

Scratches on the labial surface of the teeth were observed using a Scanning Electron Microscope, and detailed three-dimensional models of the surfaces were produced using an Alicona Infinite Focus optical surface measurement system [26–27]. The orientation of the non-masticatory striations relative to the occlusal plane was measured on scanning electron microscope images of the labial surfaces using the four orientation categories defined by Lalueza Fox and Frayer [27]: horizontal, angle extending between 0°-22° and 158°-180°; right oblique, from the individual’s upper right to lower left, angle extending between 23° to 67°; left oblique, from the individual’s upper left to lower right, 113°-157°; and vertical, 68°-112°.

## Descriptions

The six teeth are complete apart from the apices of the roots of the right-hand teeth, which are missing. The apex of the left fourth premolar root is also slightly chipped. The roots and exposed dentine of the right-hand teeth are stained black. This is less pronounced on the left-hand teeth, which have patches of black stain on the roots and none on exposed dentine. All the roots have patches of concretion. There also appears to be mineralisation towards the cervix, and extending onto the roots, causing a high gloss. The left central incisor is split near-vertically into two pieces along the mesiodistal axis of the crown and the buccal two thirds of the root. All the teeth have minor *post-mortem* vertical cracking.

**Fig 11:**
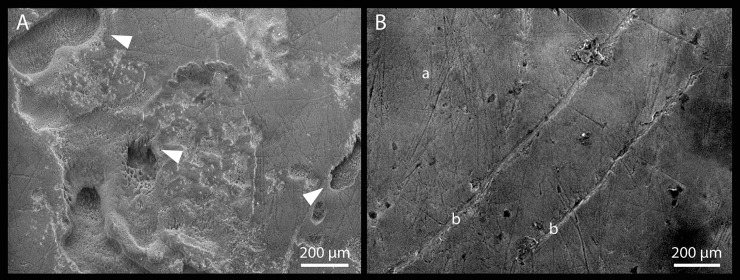
A. Scanning electron microscope (SEM) image of the labial surface of the right central incisor, showing grooves exposing the prismatic structure of the enamel (white arrows). B. SEM image of the labial surface of the left central incisor, showing microwear striations (a) and nonmasticatory striations (b).

The six teeth have suffered a taphonomic erosive process causing the partial dissolution of the enamel and the formation of obvious grooves and perforations of the crown, exposing the prismatic structure of the enamel (Fig 11A). This damage is accentuated on their roots, probably due to differences in hardness of the enamel and dentine. This type of alteration is likely to be associated with chemical degradation, similar to the dissolution of the cortical surface on bone caused by root etching [29–31]. It seems likely that the scarcity of human remains at the site is associated with this process of strong chemical degradation [32–33].

Due to *post-mortem* damage, it is not possible to ascertain the degree of *ante mortem* polish or minor chipping on the teeth. It can be said, though, that there was no major *ante mortem* chipping or fractures. The only definite *ante mortem* chipping of the enamel is along the lingual side of the exposed dentine on the right central and lateral incisors (chips 0.3 mm wide). Inter-proximal facets are visible on all teeth, apart from distally on the left central incisor, due to *post-mortem* damage, and between the right canine and right third premolar. They are flat and regularly placed except for the distal facet on the left fourth premolar, which is at the buccal end of the distal surface and oriented slightly buccally and occlusally, implying minor rotation and tilting of this tooth. There are no sub-vertical grooves on the facets.

Despite the taphonomic damage, it is still possible to observe, on the undamaged areas, a series of very fine striations on the buccal and lingual surfaces of the enamel of all the teeth (Fig 11B). These have a size, between 50 to 200 μm in length and less than 20–30 μm in width, and overlapping orientation, normally categorized as microwear. Microwear striations are assumed to be related to the mastication of food [34–36]. On the buccal surfaces of the crowns these are near horizontal on the right incisors and both vertical and oblique occluso-distal on the canine and premolars. All the teeth have vertical striations lingually and also stronger oblique striations on the canine and premolars, which are near horizontal on the fourth premolar. No striae or grooves are apparent on the mesial or distal surfaces of the teeth.

There is no indication of caries and no hypercementosis. Seen at 12x magnification, the premolars have irregular vertical corrugations on the buccal surface in the occlusal half. All the teeth have multiple disturbances of the perikymata / very mild furrow-form hypoplasia on the buccal surface towards the cervix, in particular the canine, which also shows this lingually. There is clear multiple furrow-form hypoplasia in this position on the fourth premolar (occurring at about age 5 years using Fig 96 in [37]), and this episode may be reflected in horizontal linear disturbances in the root of the third premolar near the cervix. There are two groupings of multiple furrow-form hypoplasias on the buccal surfaces of the two central incisors in the cervical half, which align (occurring at about age 3.5 years using Fig 96 in [37]). These also occur on the lateral incisor, but very faintly. These might be expected to be reflected in the occlusal part of the canine buccal surface, but this is much eroded and only a partial single furrow is visible. There is no pit hypoplasia on the teeth that can be seen at 12x magnification. There is no evidence of calculus. There are no irregularities of occlusion and there appears to have been slight overbite.

**Fig 12:**
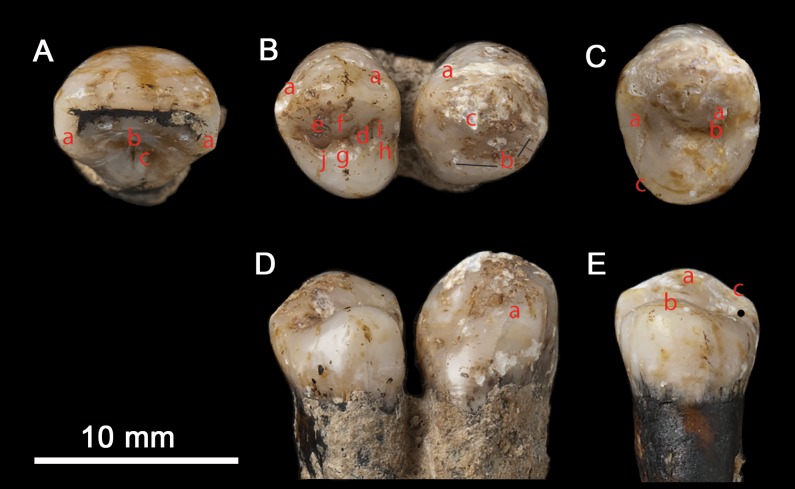
Photos of A. the occlusal surface of the right lateral incisor showing: a: shovelling; b: cingulum pit; c: tuberculum dentale. B. the occlusal surface of the right canine and third premolar showing: a: double shovelling; b: mesial canine ridge; c: distal accessory ridge; d: mesial interruption of sagittal sulcus and associated buccal and lingual mesial accessory ridges; e: buccal cusp distal accessory ridge; f: buccal cusp median ridge; g: lingual cusp median ridge; h: sagittal sulcus runs over mesial margin; i: anterior fovea; j: posterior fovea. C. the occlusal surface of the left fourth premolar showing: a: mesial and distal triangular grooves; b: bridge across junction of distal triangular groove and sagittal sulcus. D. the lingual surface of the right canine and third premolar showing: a: mesially placed lingual ridge and E. the lingual surface of the left fourth premolar showing: a: medially placed buccal cusp; b: mesially placed lingual cusp; c: concave distal margin of buccal cusp.

All the teeth have single roots that taper to a rounded point (where complete) and all have single root canals. The roots are not entirely regular in that, in most, the curvature or direction of one or more sides changes at a point one third of the length of the root from the apex. None of the teeth has a waist or prominent overhang of enamel at the cervix, or a buccal/lingual basal prominence. The measurements of the Magubike teeth are summarized in Table 10. Locations of crown morphological traits are indicated in Fig 12.

**Table 10:** Measurements of the Magubike hominin teeth (in mm to nearest 0.05).

Measurement	Direction	Upper Right Central Incisor	Upper Left Central Incisor	Upper Right Lateral Incisor	Upper Right Canine	Upper Right Third Premolar	Upper Left Fourth Premolar
Crown	MD length	9.6	9.7	8.2	7.8	7.4	7.4
Crown	BL breadth	7.7	7.75	7.1	9.1	9.5	9.35
Crown	Area (MD x BL)	74	75	58	71	70	69
	Index ([BL/MD]x100)	80	80	87	117	128	126
Root robusticity	MD	6.9	6.7	6.0	6.2	5.6	5.7
Root robusticity	BL	7.2	7.2	6.6	9.0	8.8	8.6
Root robusticity	MD x BL	50	48	40	56	49	49
Cervical	MD	7.1	7.3	6.1	6.4	5.9	6.1
Cervical	BL	7.5	74	6.9	9.0	9.0	8.9
Cervical	MD x BL	53	54	42	58	53	54
Crown height		>10.4	>11.3	>10.0	10.7	7.9	6.8
Root length		>13.5	16.5	>12.1	>18.1	>17.0	21.6
Buccal Angle	(Angle to vertical, occlusal edge to point of maximum curvature)	28	28	27	22	20	17
Root robusticity / crown area ratio		0.67	0.64	0.68	0.79	0.70	0.71

### Incisors

The wear on all three incisors is grade 4 (moderate dentine exposure), flat and lingually inclined. They all have a line of exposed dentine, approximately 7.4 x 1.2 mm on the central incisors and 5.7 x 0.7 mm on the lateral incisor. The dentine is near-flat on the right central incisor and normally worn on the other two incisors. Buccal to the dentine, the occlusal surface is rounded. Lingual to the dentine (and mesial and distal to it, where not eroded) is a lingually inclined wear facet. This is rounded lingually. All three incisors have deep scratches on the buccal surface (described below) and have slight rounding of the lingual surface due to wear.

The presence of regularly placed mesial interproximal facets on the central incisors indicates the absence of winging or midline diastema. The crown shapes are 'tapering' viewed buccally. The shape of the lateral incisor crown is normal, with no reduction or distal indentation. There is mild shovelling (grades 1–2) on all three incisors and mild double shovelling (grade 1) on the central incisors. On the central incisors the double shovelling is more pronounced mesially than distally and is barely visible distally on the right central incisor. There are single distally placed finger-like projections on the central incisors, and a grade 5 tuberculum dentale in the form of a narrow buccolingual ridge, with a cingulum pit distal to it, on the lateral incisor. There is a central vertical groove on the buccal surface of the left central incisor and on the lateral incisor.

The roots of the central incisors are straight and have a rounded triangular shape, flattest mesiolingually. The taper in the apical third of these roots is greater lingually than buccally. The root of the lateral incisor has a mesiodistally flattened and rounded rectangular shape, and a slight distal curvature towards the apex. The buccal side is distinctly longitudinally convex. There are no grooves on the root.

### Canine

Wear is grade 1 (in occlusion but no dentine exposure). There are lingually inclined wear facets on the mesial and distal margins of the canine cusp. The distal margin buccal to the facet is rounded due to wear (mesial margin eroded). There is also rounding of the lingual surface due to wear, more pronounced than on the incisors.

The occlusal shape of the canine is near circular, swollen lingually, with a mild mesiobuccal bulge. This, together with the blunt cusp shape, gives the tooth a premolariform appearance. There is no tuberculum dentale, but there is a wide, mesially placed, lingual longitudinal ridge (tubercle extension). There is mild shovelling (grade 1) and moderate double shovelling (grade 2). A canine mesial ridge is present and a distinct grade 3 distal accessory ridge.

The root is mesiodistally flattened, widest buccally. The angle of taper to the apex is greater distally than mesially in the apical part of the root. The maximum mesiodistal dimension is at the cervix, but the maximum buccolingual dimension is 4 mm from the cervix, due to the convexity of the lingual surface of the root. There is a very distinct longitudinal curvature to lingual of the buccal surface, so that the apex is placed lingually to the axis of the tooth. There is a mild vertical groove on the distal surface.

### Premolars

Wear is grade 1 (in occlusion but no dentine exposure). The buccal and lingual cusp tips of the premolars are both rounded due to wear. On the third premolar there are wear facets on the distal margins of both cusps, both inclined lingually. These are also present on the fourth premolar, as well as a lingually inclined facet on the mesial margin of the buccal cusp, which continues onto the ridge across the sagittal sulcus.

The occlusal surface of the premolars has a regular rounded-triangle shape. The buccal cusps are medially placed, but there is pronounced mesial placement of the lingual cusp in relation to the buccolingual longitudinal axis of the tooth at the cervix (least on the third premolar). Both cusps have strong margins linking to prominent mesial and distal marginal ridges. These are straight, apart from the distal margin of the fourth premolar buccal cusp, which is concave. The sagittal sulcus continues u-shaped into the distal triangular groove. On the fourth premolar there is a slight enamel bridge at this point. The indistinct distal fovea is at the most lingual point of the sagittal sulcus. The sagittal sulcus does not interrupt the mesial or distal margins, rather goes over the mesial margin on the third premolar. Both triangular grooves are fissured. The buccal essential crest is wide and prominent on both premolars and the lingual essential crest is prominent on the third premolar, but slight on the fourth premolar. On both premolars there are mesial and distal accessory occlusal ridges buccally, and mesial lingually. The mesial accessory ridges on the buccal and lingual cusps of the teeth link to form a mesially placed low ridge (transverse crest) over the sagittal sulcus. This does not reach the cusp tips and is weaker on the third premolar. On both teeth, buccal and lingual grooves ascend the distal side of this ridge from the sagittal sulcus. On the third premolar there is a strong ridge between these two grooves. There is also a slight interruption of the sagittal sulcus between the buccal and lingual essential crests on the third premolar. The anterior fovea is buccolingually elongated (the mesial triangular groove). There is no wrinkling of the occlusal surface. The wide canine groove on the third premolar is very distinct. Both premolars have double shovelling (grade 2 mesially and 1 distally on the third, and the reverse on the fourth), and on the fourth premolar the distal double shovelling has multiple vertical grooves.

The roots are long and slender and mesiodistally flattened, and the root of the third premolar has a slight distal curvature towards the apex. The complete root of the fourth premolar is similar in certain respects to that of the canine ― greater angle of taper distally, longitudinal convexity of the lingual surface towards the cervix, and longitudinal curvature to lingual of the buccal surface in the apical half, but not as pronounced as in the canine. Both premolar roots have distinct infolding forming wide grooves from approximately 3.5 mm to 9 mm from the cervix, mesial and distal on the fourth premolar (the distal being the deepest), and distal on the third premolar.

## Results and discussion

### Number of individuals

Although only two of the teeth are linked by alveolar bone, it is reasonable to assume that they all come from the one individual, despite the fact that they were found in two groups, 10 cm in depth apart: -

- The interproximal facets on adjacent teeth fit;
- The levels of wear are consistent with this assumption;
- The episodes of furrow-form hypoplasia align on the incisors and on the premolars;
- There is a similarity of size, shape and morphology between the two central incisors and between the two premolars; and
- Each of the two groups in which the teeth were found contained one of the central incisors, these almost undoubtedly being antimeres.

However, the fact that the teeth were found at different levels suggests that there has been a certain amount of mixing and/or rapid accretion of the deposits at the site.

### Age at death

Smith and co-authors [38–39] have demonstrated, using synchrotron virtual histology, that the Middle Palaeolithic *Homo sapiens* juveniles from Qafzeh and Jebel Irhoud had similar dental development times to those of modern humans. However, it should be noted that Guatelli‐Steinberg and Reid [40] found that the perikymata distribution pattern in Qafzeh anterior teeth differed from recent human teeth in the same way as found in Neanderthals and considered this to be the retention of an archaic trait. Smith *et al*.'s [38–39] findings suggest that it is reasonable to use modern human criteria to age both archaic and anatomically modern humans from the later middle Pleistocene and the late Pleistocene. Apical closure of the fourth premolar (the last of the teeth present to become fully developed) is complete, giving a minimum age of 14 years (Table 5.1 in reference [41]). There is little wear and no dentine exposure on the canine or premolars (dentine exposure on the canine appears to be imminent), suggesting that the teeth are from a young individual. Thoma and Vallois [42] considered that the archaic *H*. *sapiens* ‘Rabat Man’, with a similar (but slightly lower) level of wear, was aged 14–15 years based on the degree of third molar development, using modern human criteria.

### Wear

The lingual rounding of the Magubike incisors and canine is unlike LSAMAT (lingual surface attrition of the maxillary anterior teeth) described by Turner and Cheuiche Machado [43] and also unlike the irregular cupped wear produced by erosion [122], but rather it and the prominent and largely near horizontal lingual striations on the canine and premolars are consistent with lengthy mastication of vegetable material [123–124].

Grine *et al*. [44] and Henshilwood *et al*. [45] note that the presence of faint horizontal striae on the mesial and distal surfaces of premolar and molar crowns is common amongst South African MSA teeth, occurring at Blombos Cave, Klasies River, Die Kelders Cave, Border Cave, and Equus Cave. As stated above, these are not apparent on the Magubike teeth.

### Morphology

It is difficult to make comparisons and to look for trends in either morphology or measurements of relevant MSA teeth, since there is little African Pleistocene comparative material available for the preserved Magubike teeth, particularly in sub-Saharan Africa, and much of the material that is available is very worn (e.g. Nazlet Khater, Broken Hill, Lake Eyasi, Herto, Ngaloba, and Kabua). The morphological traits of the Magubike teeth are presented, along with comparative data, in Tables 11 and 12.

**Table 11:** Morphology comparative data, upper incisors and canine, percentage frequencies.

Trait	Magubike Left	Magubike Right	Early Modern Afro-Asian	African Mid / Late Pleistocene	Kenyan early Holocene	Recent Bantu	Recent San
**Central Incisor**							
Bilateral winging	No	No		0 (0/8)	0 (0/3)	4.5 (178)	16.7 (90)
Crown shape tapered	Yes	Yes		67 (2/3)	67 (2/3)	12.0† (100)	
Shovelling >1	1	1	33 (2/6)	40 (2/5)	50 (1/2)	23.2* (181)	40.0* (80)
Double shovelling >1	1	1	0 (0/7)	25 (1/4)	33 (1/3)	1.7 (177)	0.0 (79)
Occlusal labial convexity >1	1	1	50 (4/8)	40 (2/5)	33 (1/3)	52.8 (178)	66.3 (80)
Tuberculum dentale (inc. finger-like projections)>1	1	2	50 (3/6)	100 (4/4)	50 (1/2)	40.0 ˟ (214)	
**Lateral Incisor**							
Crown shape, peg/reduced		No		14 (1/7)	40 (2/5)	8.1 (86)	11.2 (178)
Shovelling >1		2	83 (5/6)	50 (4/8)	20 (1/5)	14.3 ˟ (322)	38.6 ˟ (109)
Double shovelling>1		0	0 (0/7)	14 (1/7)	40 (2/5)	26ᶲ (9/34)	
Interruption groove		No		0 (0/6)	0 (0/5)	11.6 (173)	15.7 (83)
Cingulum pit		1		0 (0/6)	0 (0/5)	4ᶲ (1/28)	
Tuberculum dentale (inc. finger-like projections)>1		5	67 (4/6)	100 (7/7) (6 x gd 5-to 6)	60 (3/5) (gd 5-to 6)	27.3 (172) (11.1˟ gd 5-to 6)	44.3 (79)
**Canine**							
Occlusal shape round		Yes		10 (1/10)	50 (3/6)	9ᶲ (5/53)	
Occlusal mesiobuccal bulge		Slight		70 (7/10)	80 (4/5)	67ᶲ (32/48)	
Shovelling >1		1	100 (5/5)	50 (4/8)	17 (1/6)	30ᶲ (13/44)	
Double shovelling >1		2	13 (1/8)	60 (6/10)	40 (2/5)	43ᶲ (21/49)	
Buccal basal prominence		No		29 (2/7)	17 (1/6)	14ᶲ (7/51)	
Tuberculum dentale >4		0	20 (1/5)	78 (7/9)	20 (1/5)	68ᶲ (30/44)	
Lingual longitudinal ridge (tubercle extension)		Mesial		75 (6/8) 3 mesial	100 (6/6) 0 mesial	91ᶲ (39/43) 21 mesial	
Canine mesial ridge >0		Yes	0 (0/5)	0 (0/8)	20 (1/5)	9.9 (172)	35.1 (77)
Distal accessory ridge >0		3	100 (2/2)	80 (4/5)	80 (4/5)	>1, 57.3 (171)	>1, 20.3 (69)
Root buccal vertical curvature		Yes		25 (1/4)	0 (0/3)	7ᶲ (4/57)	

Figures for Early Modern Afro-Asian (rounded) from Bailey [15]; Bantu, San: Unmarked Irish[115],

*Irish (personal communication),

˟Jacobson [54],

†Middleton Shaw [57],

ᶲRecent sub-Saharan East/South African sample.

Number with trait over number in sample, or number in sample, in brackets.

**Table 12:** Morphology comparative data, upper third and fourth premolars, percentage frequencies.

Trait	Right Third Premolar -Magubike	Right Third Premolar–African Mid / Late Pleistocene	Right Third Premolar–Kenyan early Holocene	Right Third Premolar–Recent East/South African	Left Fourth Premolar—Magubike	Left Fourth Premolar–African Mid / Late Pleistocene	Left Fourth Premolar—Kenyan early Holocene	Left Fourth Premolar—Recent East/South African
Accessory marginal cusp–M	No	29 (2/7)	20 (2/10)	5 (3/57)	No	25 (2/8)	20 (2/10)	10 (5/52)
Accessory marginal cusp–D	No	17 (1/6)	30 (3/10)	11 (6/57)	No	29 (2/7)	36 (4/11)	19 (10/52)
Double shovelling–M >1	2	50 (5/10)	40 (4/10)	62 (37/60)	1	60 (6/10)	11 (1/9)	7 (4/57)
Double shovelling–D >1	1	40 (4/10)	0 (0/10)	3 (2/59)	2	30 (3/10)	10 (1/10)	14 (8/57)
Buccal basal prominence	No	22 (2/9)	60 (6/10)	22 (13/58)	No	9 (1/11)	45 (5/11)	5 (3/58)
Buccal accessory occlusal ridge-M	Prominent	43 (3/7)	20 (2/10)	15 (8/55)	Prominent	80 (8/10)	60 (6/10)	53 (30/57)
Buccal accessory occlusal ridge-D	Prominent	67 (4/6)	30 (3/10)	41 (24/58)	Mild	44 (4/9)	55 (6/11)	55 (30/55)
Buccal essential crest divided	No	0 (0/6)	20 (2/10)	23 (14/60)	No	11 (1/9)	0 (0/11)	14 (8/59)
Sagittal sulcus interrupted (transverse crest)	Mesial	0 (0/7)	20 (2/10) 0 mesial	9 (6/67) 5 mesial	Mesial	56 (5/9) 3 mesial	33 (4/12) 2 mesial	16 (10/62) 8 mesial
Number of roots > 1	1	78 (7/9)	100 (4/4)	82.6† (84)	1	56 (5/9)	40 (2/5)	59.2† (83)
Number of roots >1 (San)				20. 0˜ (15)				

Number of roots:

˜Irish [116]

†Middleton Shaw [57]. Number in sample, or number with trait over number in sample, in brackets. M: Mesial; D: Distal.

#### Relationships between comparative samples

Scott and Turner [20] identified a sub-Saharan complex of morphological traits occurring amongst recent humans. They subdivided this into West Africa, South Africa, and San. Some traits specific to the teeth being studied have high or low occurrence rates in recent sub-Saharan Africans compared to other groups: -

1. Low: winging, shovelling in upper central incisors at grades above 2, double shovelling in upper central incisors, interruption grooves in upper lateral incisors.

2. High: mesial canine ridge in upper canine, two-rooted upper third premolars (though this varies between groups).

These findings are reflected in the African mid/late Pleistocene and Kenyan early Holocene samples. The Pleistocene and early Holocene samples are both characterised by low grades of incisor shovelling and double shovelling, below grades 4 and 3 respectively, and mild finger-like projections (as distinct from tuberculum dentale), grades 1 or 2. The grades of labial convexity of the central incisor are also low, below grade 3, and interruption grooves are absent. Differences in the Pleistocene sample compared to the early Holocene and recent samples used for comparison are: -

- Incisors: there are higher occurrence rates of finger like projections / tuberculum dentale and, in the lateral incisors, grades >4.
- Canine: canine mesial ridge is absent (and also absent in the early modern Afro-Asian sample). (Nevertheless, canine mesial ridge occurs at a high rate in Neanderthals [16] and Stringer *et al*. [46] considered it to be primitive for modern humans).
- Premolars: double shovelling is commoner distally on third premolars, and mesially and distally on fourth premolars; sagittal sulcus is not interrupted on third premolars, but more frequently interrupted on fourth premolars and, in third premolars, buccal accessory occlusal ridges are commoner.

The Kenyan early Holocene sample differs from both the mid/late Pleistocene sample and recent comparative samples in occurrence rates of certain traits: lateral incisor reduced, round occlusal shape of canine, buccal basal prominence in premolars (all higher) and canine tuberculum dentale (grades >4) (lower).

Certain patterns are clear in the premolar morphological traits scored: -

- Accessory marginal cusps: in all but one sample these are commoner on the distal margin than on the mesial, and cusps on the distal margin occur more frequently on P^4^ than on P^3^
- Double shovelling: this is commoner mesially than distally on the P^3^, as found by Scott and Turner [19] in anterior teeth, but there is no clear pattern on the P^4^.
- Buccal basal prominence: most often found on the P^3^
- Buccal accessory occlusal ridges: Burnett et al. [126] found these to be commoner on the P^4^ than on the P^3^ and commoner distally than mesially on the P^3^. This generally holds here
- Sagittal sulcus interrupted: most often found on the P^4^.

#### Relationship of Magubike teeth to comparative samples

The Magubike teeth fit well with recent sub-Saharan teeth and the mid/late Pleistocene sample in not having traits that only occur at a low level, such as winging, interruption groove, and reduced lateral incisor. In addition, there is the absence of high grades of shovelling and double shovelling in the incisors and the presence of only low grades of finger-like projections and labial convexity. Magubike contrasts with the Pleistocene sample primarily in the canine ― round occlusal shape, lack of tuberculum dentale at grade >4 and presence of canine mesial ridge ― and also in the interruption of the sagittal sulcus in the third premolar (though this last trait is also rare in the recent East/South African sample). In this respect, it is in better accordance with the Kenyan early Holocene sample, but sample sizes are small. The presence of single roots on the Magubike third premolar contrasts with both the Pleistocene and the early Holocene samples, but not with the Gwisho sample, where two of three teeth are single rooted. There are cases where there is a clear difference between the Pleistocene and early Holocene samples on the one hand, and the recent comparative samples on the other, and where the Magubike teeth are in accord with the earlier teeth:

- Grades of tuberculum dentale above 4 in lateral incisors (present at Magubike) occur more frequently in both the Pleistocene and the early Holocene samples than in the recent samples. In the recent sub-Saharan East/South African sample only two of thirty-four teeth (6%) have a tuberculum dentale > 4.
- This also applies to the tapered shape of central incisors where, in addition, eight of ten Gwisho central incisors are tapered.

The cingulum pit found on the Magubike lateral incisor only occurs on one Gwisho tooth, and one tooth in the recent East/South African sample, of the material studied (though, in the latter, six teeth had calculus or concretion obscuring the site), but can be a rare feature, e.g. none were found in a Neolithic population from Çatalhöyük in Turkey [46]. The occurrence of grade 2 double shovelling, mesially on the third premolar and distally on the fourth premolar at Magubike, mirrors the occurrence rates for this trait found in the recent East/South African sample. Other minor traits found at Magubike, such as concave shape of premolar buccal cusp margins and an enamel bridge (interruption) between the sagittal sulcus and a triangular groove on a premolar, are found in both the Pleistocene and the early Holocene samples as well as in the recent East/South African sample.

The mesial placement of the canine lingual longitudinal ridge seen at Magubike is common in the mid/late Pleistocene and recent East/South African samples, and at Gwisho (2/3), but does not occur in the Kenyan early Holocene sample. Interruption of the sagittal sulcus is more frequently found in the fourth premolar than in the third in all three reference samples in Table 12, and when it occurs it is often placed mesially, as at Magubike.

The pronounced buccal vertical curvature of the canine root found at Magubike is seen in early teeth, *Homo erectus* and Neanderthal [22, 47], but not in samples of early modern *Homo sapiens* (Skhūl and Qafzeh) or European Upper Palaeolithic *Homo sapiens* [48]. In recent teeth, the buccal surface is usually straight or has a mild curvature [49]. The few canine roots that could be observed in the Kenyan early Holocene sample, and the two in the Gwisho sample, are straight, and in the mid/late Pleistocene sample only the Broken Hill canine has similar curvature to that of the Magubike canine. However, four instances were found in the recent sub-Saharan East/South African sample (7%). Similarly, the incomplete root of the Magubike lateral incisor has pronounced buccal vertical curvature in contrast to the more evenly tapered form of recent teeth [50]. A lateral incisor of this form occurs at the Klasies River Main site [51] and in one of four in the Gwisho sample, but in just one out of seventy-five in the recent East/South African sample.

#### Middle Pleistocene traits

Bermúdez de Castro [52] identified certain traits found in middle Pleistocene hominin teeth ― mesiobuccal bulge viewed occlusally (*tuberculum molare*) in the upper third premolar; buccal cingulum on upper (and lower) canines and premolars; secondary crenulation and fissuration (wrinkling) of posterior teeth. In the mid/late Pleistocene sample these traits are only found in certain *Homo heidelbergensis* / archaic *Homo sapiens* teeth and some Aterian teeth. They do not occur in the Kenyan early Holocene or Gwisho samples studied or at Magubike.

1. Premolar asymmetry (viewed occlusally) (*tuberculum molare*).
  The mesiobuccal bulge of the upper third premolar, also seen in some *Homo erectus* specimens [23], occurs at Salé and at Eyasi. Higham *et al*. [42] observed this trait in samples from Skhūl and Qafzeh (25%) and European Upper Palaeolithic (8%), but not in recent European teeth.
2. Cingulum (horizontal enamel shelf on buccal/lingual surface).
  Cingula on upper canines and premolars, described by Briggs [52] as vestiges of cingula in the form of buccal and lingual swellings at the base of the crown, are found at Rabat and Mugharet el ‘Aliya.
3. Premolar wrinkling (multiple additional or distorted occlusal ridges).
  Occlusal wrinkling of upper premolars occurs at Rabat. Of the later teeth, premolar wrinkling also occurs at the Holocene site of Wadi Halfa.

#### Possible San affinities

As stated above, Scott and Turner [20] subdivide the sub-Saharan complex of morphological traits into West Africa, South Africa and San. Of these three sub-complexes a number of characteristics in the Magubike teeth suggest possible San affinities: mild shovelling of the incisors, the tapered shape of the buccal surfaces of the central incisors, the round premolariform occlusal shape of the canine and the presence of a mesial canine ridge, and the single rooted third premolar. None of these are specific to San, though they tend to be more common in San than in other sub-Saharan Africans. Of the traits for which figures are published for recent groups (Tables 9 and 10 or as stated): -

- upper incisor shovelling: for any level of expression in the central incisor the San sample has, at 44.2%, approximately three times the percentage rate of Bantu (15.4%) [53]. Semi (grade 2) shovelling and above in lateral incisors occurs in 38.6% of the San sample against 14.3% of Bantu
- canine mesial ridge: occurs in 35.1% of the San sample against 9.9% for Bantu and 12% for South African excluding San [20].
- single rooted upper third premolars: in San these are the norm at 80% against 17.4% in Bantu and 37% for South African excluding San [20].

Oranje [54] described Bushman upper central incisors as being fan shaped (i.e. tapered) giving an increase in mesiodistal diameter from the enamel level to the cutting edge. He also referred to the upper canine as not being peg shaped, but instead resembling the premolar. Galloway [55] also describes Bushman upper incisors as being Type 2 (tapered), in contrast to the Bantu incisor, which is usually Type 3 (ovoid) [57] and refers to the premolariform nature of the upper canine as being a specialised manifestation of the San dentition. Against this, the canine distal accessory ridge at above grade 1, present at Magubike, occurs at a lower frequency in San (20.3%) than is found in Bantu (57.3%).

In summary, the Magubike teeth have the characteristics typical of African sub-Saharan *Homo sapiens* Holocene teeth, but aspects of the morphology of the upper canine and third premolar are atypical for the African mid/late Pleistocene sample. However, this may be at least partly due to its North African bias. Certain traits present ― e.g. the root shape of the lateral incisor and canine, and the presence of a cingulum pit on the lateral incisor ― are rare or absent in the comparative samples. Traits specific to *Homo heidelbergensis* / archaic *Homo sapiens* and Aterian teeth are not present. Despite their large size, there is some suggestion of San affinities, though this interpretation must be very tentative, being based on a single individual.

### Measurements

The measurements of the Magubike teeth are summarised in Table 10, and references for the comparative samples (Tables 6, 7 and 8) are presented in Table 5.

#### Crown measurements (Table 6)

**Fig 13:**
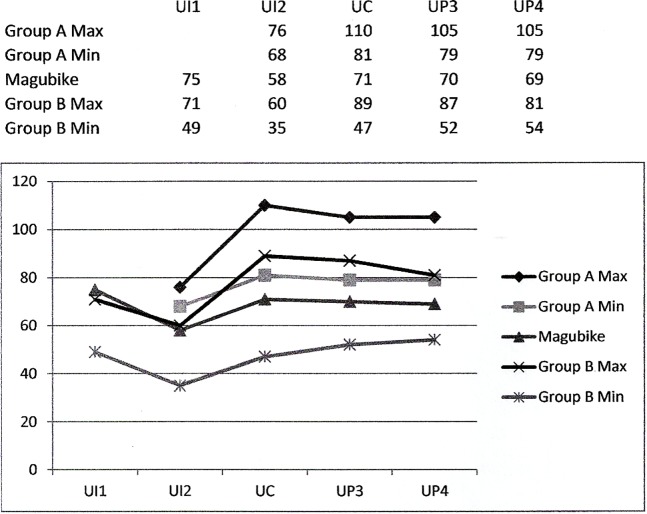
Crown area comparisons, Group A: African *H*. *heidelbergensis*, archaic *H*. *sapiens*, Aterian. Group B: sub-Saharan Middle Stone Age to mid Holocene *H*. *sapiens*.

For the teeth studied, there is a difference in size (crown area) between the large *Homo heidelbergensis /* archaic *Homo sapiens* teeth (e.g. Thomas Quarries III, Salé, Rabat, Jebel Irhoud, Broken Hill, Lainyamok, Wadi Dagadlé, Eyasi) and the North African Aterian teeth (Mugharet el ‘Aliya, Dar es Soltan, Smuggler's Cave) on the one hand, and later North and sub-Saharan African *Homo sapiens* teeth on the other. The Magubike teeth clearly belong to the second group (Fig 13). The central and lateral Magubike incisors are large compared to contemporary and later material, but the crown areas and crown indices of the canine and premolars all lie within the ranges of values of the small sample of sub-Saharan Middle Stone Age *Homo sapiens* teeth. The crown dimensions of all the Magubike teeth fit in the wide range of values found in the recent Bantu sample. The size relationship between the two premolars varies in different samples and specimens but generally the third premolar is a little larger than the fourth as it is at Magubike.

The crown index (shape) figure of the Magubike central incisors varies in its relationship to the means of African samples in the second group described above, though that of the canine is higher than all the sample means in this group and those of the lateral incisor and both premolars (with one exception) are lower. Again, however, all the Magubike crown indices fit in the range of values found in the recent Bantu sample.

#### Crown height (Table 7)

Teeth included in Table 7, other than those marked ">", have minimal wear. In the comparative data, the crown heights of the Rabat and Aterian Mugharet el ‘Aliya teeth are particularly large and those of the San Bushmen are notably small. The Magubike fourth premolar crown height is small compared to other material, and the large difference in crown height between the Magubike third and fourth premolars is only seen in the *Homo erectus* specimens and at Rabat (all greater). The Magubike third premolar and canine crown heights are intermediate compared to other *Homo sapiens*, and the crown heights of the incisors are large, considering the level of wear, which is equivalent to a likely loss of crown height of at least 2 mm (the level of wear on the Rabat and Magubike lateral incisors being similar).

#### Root length (Table 8)

The actual/estimated Magubike root lengths are long compared to other anatomically modern material (other than Skhūl) listed in Table 8, being above or top of range, in particular those of the premolars, which are longer than in all the other *Homo sapiens* samples. The Rabat and Sea Harvest premolar roots are similarly long compared to more recent African material, above range for all samples except Bantu. Incisors from an LSA skeleton, indirectly dated at 11–16 ka, from the nearby Mlambalasi rockshelter have particularly long roots too—17.1 mm and 17.8 mm for central and lateral incisors respectively [58]. The estimated equal lengths of the Magubike central and lateral incisor roots, and the greater length of the fourth premolar root compared to the third, is reflected in the means of the recent African samples.

In summary, the crown areas of the Magubike teeth imply that they are not *Homo heidelbergensis* / archaic *Homo sapiens*. The teeth are notable for the large size of the incisor crowns, the low crown height of the fourth premolar, the difference in crown height between the third and fourth premolars, and the long roots, particularly of the premolars.

### Surface scratches

**Fig 14:**
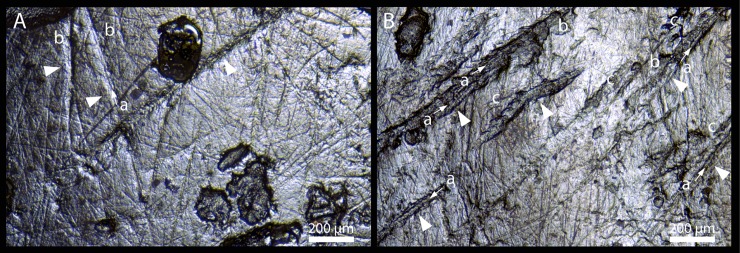
Images of non-masticatory scratches on the labial surface of left central incisor obtained using the Alicona 3D Infinite Focus microscope. White triangles indicate nonmasticatory scratches, and a, b, c show features consistent with incisions made by a stone tool edge: a, internal micro-striations; b, Hertzian cones; c, shoulder effect (micro-striations along one or both edges of the incision).

**Fig 15:**
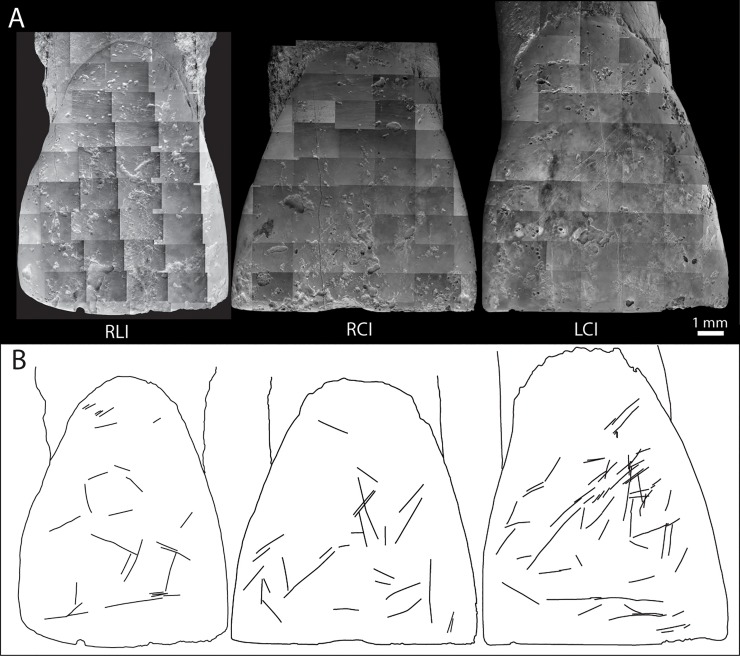
A. Composite of SEM images using a LEO1455VP scanning electron microscope of the right lateral incisor (RLI), the right central incisor (RCI) and the left central incisor (LCI). B. Outline drawings showing masticatory scratches on the labial surface of RLI, RCI and LCI.

The three incisors are marked on the labial crown by scratches much coarser than microwear striations, none of which have been observed on their lingual crown. These scratches are much longer and wider than would have been produced during mastication, so they are defined as non-masticatory (Fig 11B; [52, 59–65]). The non-masticatory scratches observed on the Magubike incisors are characterized by microscopic features consistent with incisions made by a stone tool edge (Fig 14A and 14B): internal and lateral micro-striations, Hertzian cones, and a ‘shoulder’ effect along one or both edges [26–27, 59–60, 66–70]. The left central incisor is the more marked by these coarser scratches, mainly concentrated on the central and lateral part of its labial face. The right central incisor and the right lateral incisor present fewer scratches, scattered through the central and lateral portions of the labial faces of the teeth (Fig 15A and 15B).

Non-masticatory striations have been previously reported in some fossil hominins from middle and late Pleistocene sites such as Boxgrove, UK [65], Mauer, Germany [61], Atapuerca Sima de los Huesos, and Cova Negra, Spain [55, 71], Krapina and Vindija, Croatia [27, 71] and other Neanderthals [131] as well as in recent populations [64, 73]. Partly matching the deep scratches occurring on the buccal surfaces of the Magubike incisors, Grine *et al*. [74] and Henshilwood *et al*. [42] describe a series of deep, short, sub-parallel striae running transversely on the labial surface of an upper deciduous central incisor from Blombos Cave (South Africa), which are comparable in terms of width (12–24 μm) to the scratches observed on Neanderthal incisors, but dissimilar in being predominantly horizontal rather than oblique and being most numerous in the cervical half of the labial surface. These scratches have been generally interpreted as ante-mortem cut-marks produced by stone tools used to cut food and/or other materials held between the jaws. The tools may have occasionally penetrated the material causing the scratching of the teeth. Scratches on the Magubike teeth are characterised by a wide distribution across much of the labial surface, and overlapping relationships, which suggest that use of the front teeth as tools included regularly repeated activities undertaken throughout the life of the individual.

The orientation of scratches on incisors has often been associated with handedness [28, 52, 72–73, 75]. Striations on the Magubike incisors are predominantly right oblique, with higher values for horizontal striations on the left central and the right lateral incisors (no statistical difference has been observed between the three teeth (Figs 14 and 15). The “right-oblique” orientation has been more frequently observed on fossil specimens in other studies and has been interpreted as evidence of right handedness [28, 52, 72]. The predominant “right-oblique” orientation for the Magubike incisors, more obvious for the two central incisors, may suggest a right-handed tool user cutting obliquely. Nevertheless, the limited number of scratches and the heavy *post-mortem* erosive damage of the enamel do not allow for a definitive association of these scratches with handedness.

## Concluding remarks

The discovery of MSA teeth at Magubike was unexpected. Human skeletal remains are rare in both MSA and Pleistocene Later Stone Age (LSA) contexts in East Africa [58, 76], while Holocene LSA ones are more common. In earlier literature, East African Holocene skeletal remains are described as coming from individuals who were tall and linear, while South African ones are generally small [76–77]. Small bodied individuals recovered from East African LSA sites have often been described as having “Khoisanoid affinities”, implying an assumed connection with the small bodied people of South Africa, including the San [75]. There are also still people in northern Tanzania today whose languages are often related to Khoisan (the Hadzabe and Sandawe), implying some possible past connections with southern African peoples [78].

A fragmentary human skeleton was excavated from a late Pleistocene LSA context at Mlambalasi rockshelter in 2010, not far from Magubike [58, 76]. Twenty-six of the teeth of this individual were recovered, along with numerous fragmentary postcranial elements. The bones had no collagen, so could not be directly dated, but associated material (one charcoal sample, two *Achatina* snail shells and two ostrich eggshell beads) have been radiocarbon dated between approximately 11,000 and 16,000 years ago [76]. Sawchuk’s [58] analysis of this skeleton emphasized its small body size and small teeth (the crown dimensions of which are, with one exception, within or below the ranges of value for length and breadth given by Drennan [79] for San Bushmen), in light of debates about possible San affinities of East African individuals. She also noted that it did fit the general patterns of body size documented in other studies of East African LSA remains. But, since most of these belong to the Holocene, there could be a different pattern in Pleistocene individuals [58, 76].

The six Magubike teeth are differentiated from African archaic *Homo sapiens / Homo heidelbergensis* and Aterian teeth in terms of size and the lack of morphological traits that could be associated with these groups. They are also differentiated from the Kenyan early Holocene and Gwisho samples and more recent teeth in terms of the relative size of the incisors, the incisor crown heights, the relative crown heights of the two premolars, and the long premolar roots. However, the morphological evidence is contradictory, principally in relation to the canine having traits that are rare or absent in the African mid/late Pleistocene sample, though this may be partly due to the small size of this sample and its North African bias.

These teeth are important because of the rarity of East African MSA human teeth combined with their relatively minimal wear. They all come from the upper jaw and represent an MNI of a single individual. When compared to those from other African (and non-African) samples, they reveal relationships to early modern *Homo sapiens*. This is consistent with a minimum age of at least 45,000 years old and their MSA archaeological context. The teeth are also significant in showing greater signs of San-like traits than most other MSA-associated materials. Given recent genomic studies suggesting a much greater time depth for San genetic lineages [129–130], such traits may be indicative of deep connections in time, but more fossil data are clearly required. Magubike is the first site in southern Tanzania that has produced a long MSA sequence associated with organic materials (snail shells, mammal bone and ostrich eggshell beads), let alone human remains. The incisors show evidence of cultural modification related to the use of the mouth as a third hand. Test pit 3, the unit from which the hominin teeth derive, contains a recent deposit, below which is 1.6 metres of sediment with MSA artifacts. Three ostrich eggshell beads or bead preforms from the MSA levels of test pit 12 (adjacent to test pit 3) date to between 31,000 years BP and the effective radiocarbon limit of 50,000 years ago [14]. These come from between 50 and 100 cm below the surface. The lithic assemblage from test pit 3 includes Levallois points and further excavations in the vicinity reveal assemblages that could belong to the early MSA. However, the presence of late Pleistocene beads as well as the shell radiocarbon dates lead to the conservative conclusion that the deposit from which the teeth derive is at least 45,000 years old. The possibility that the teeth are from the late MSA is supported by their possible San attributes, features which are as yet only recognised from the late MSA and early LSA (e.g. Border Cave). Further excavations at Magubike and at related sites in Iringa [80, 81] should help clarify the status of these MSA teeth and will perhaps provide other fossil human material.

## Supporting information

S1 File Additional information on ESR methods. (DOCX)
