# Supplementary material for: Middle Stone Age human teeth from Magubike rockshelter, Iringa Region, Tanzania
Source: PLoS One. 2018 Jul 31;13(7):e0200530. doi: 10.1371/journal.pone.0200530 (PMC6067719; doi:10.1371/journal.pone.0200530)
Supplement: S1 File — (DOCX) [file pone.0200530.s001.docx]

**Supplemental Information**

Additional information on ESR methods:

1. The fitting function is single saturating exponential, with 1/I^2^ weighting
2. The fitting program is Vfit.
3. Two programs have been used for age calculations: Data-HPS and ROSY. The differences between them are less than the experimental uncertainty. Beta attenuation factors are built into the programs, not calculated separately.
4. The alpha efficiency factor for teeth is 0.13 ± 0.02
5. The dose conversion factor for the gamma irradiator is 0.88.

Figure Captions:

SI Fig A: Typical spectrum of tooth enamel from Magubike tooth.

SI Fig B: Growth curve of Magubike sample (PT83en1) using Vfit. The maximum added dose is 7 times the AD. Reducing the maximum added dose to 5 times the AD did not change the calculated age within experimental uncertainty.

SI Fig A: Typical spectrum of tooth enamel from Magubike tooth.


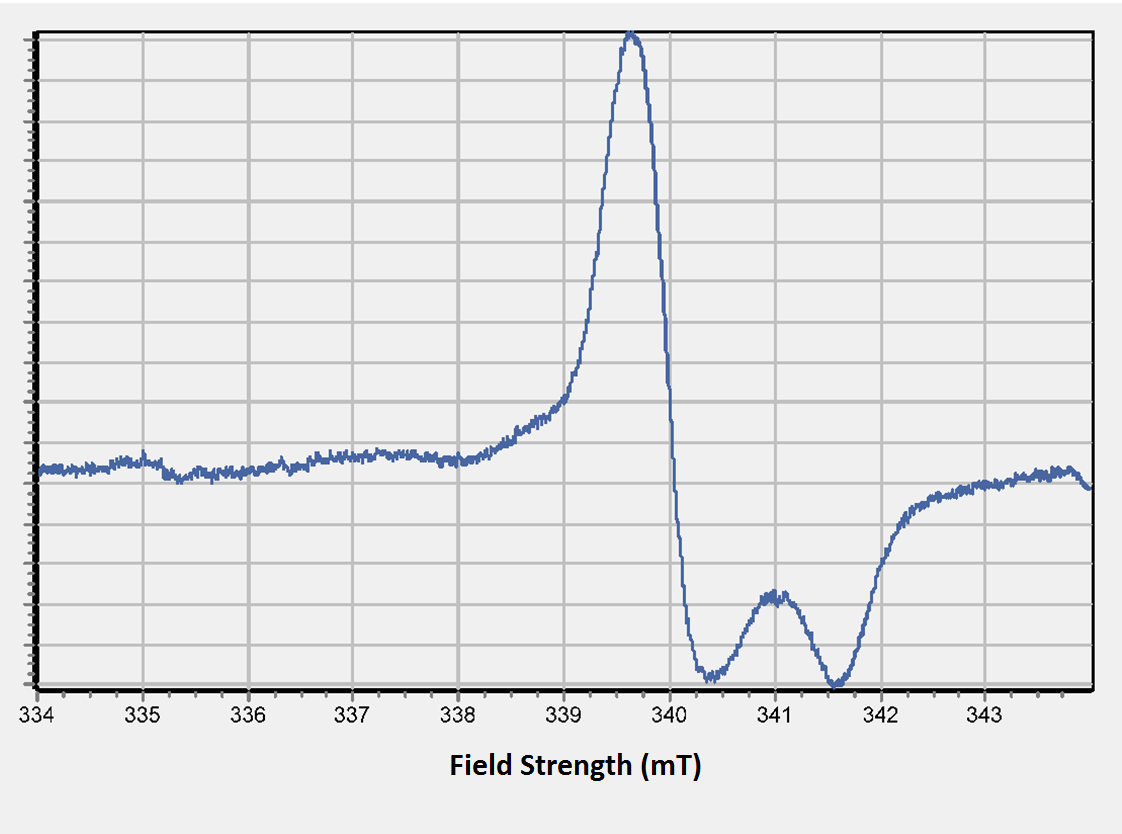


SI Fig B: Growth curve of Magubike sample (PT83en1) using Vfit. The maximum added dose is 7 times the AD. Reducing the maximum added dose to 5 times the AD did not change the calculated age within experimental uncertainty.


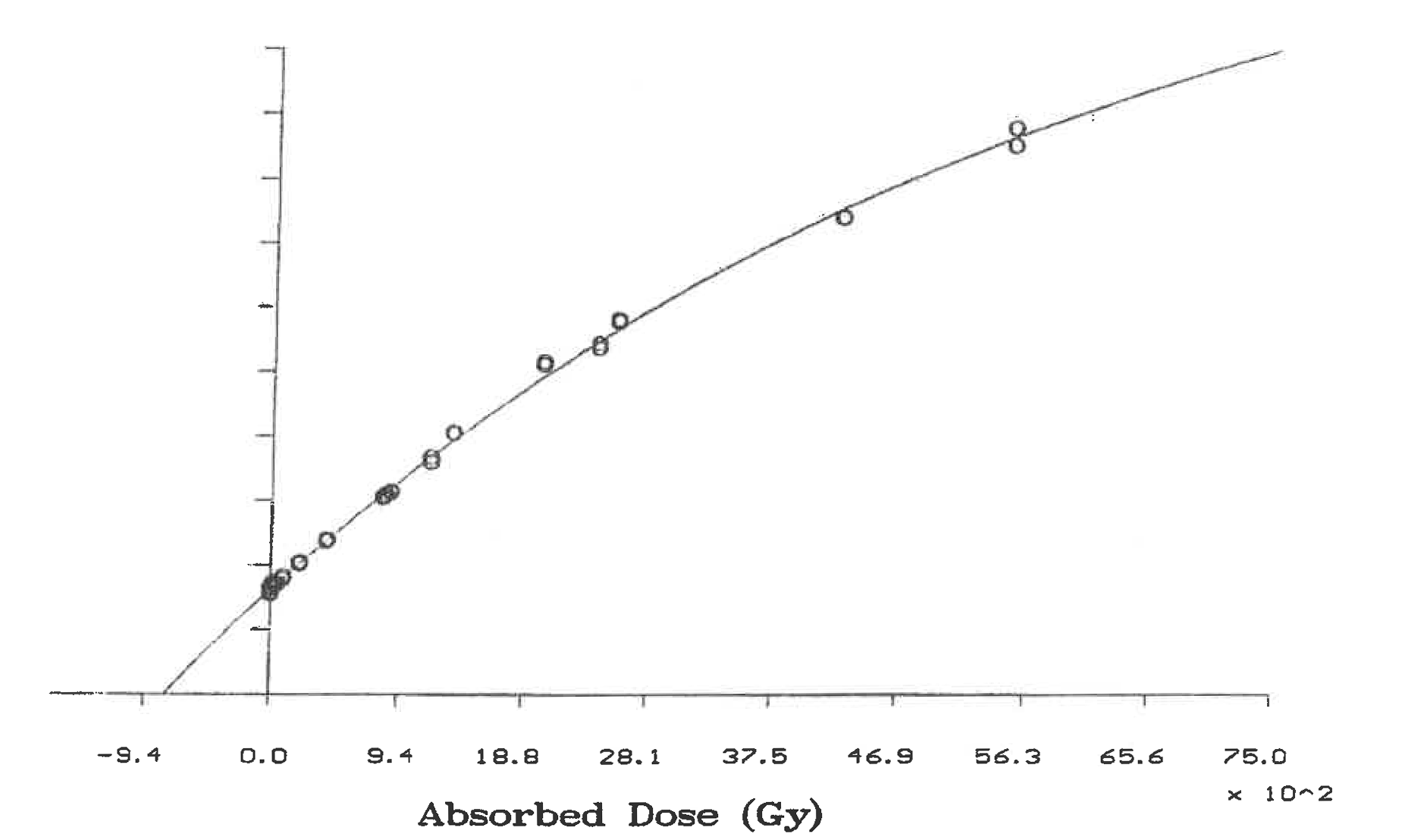


**Supplementary Table A. Sedimentary dose rates from Magubike: water content: 10% ± 5%. Average value (excluding solid rock values): 4.120 ± 0.298 mGy/a.**

| **Sample** | **TP** | **Depth** |  | **U (ppm)** | **Th (ppm)** | **K (%)** | **D(ext) mGy/a** |
| --- | --- | --- | --- | --- | --- | --- | --- |
| MAG44b | 9 | 50-60 |  | 9.48 | 103.23 | 3.90 | 6.571 |
|  |  |  | ± | 0.02 | 1.65 | 0.10 | 0.416 |
| MAG45b | 9 | 50-60 |  | 5.67 | 39.90 | 3.69 | 3.211 |
|  |  |  | ± | 0.02 | 0.63 | 0.10 | 0.202 |
|  |  |  |  |  |  |  |  |
| MAG50c | 12 | 80-90 |  | 8.79 | 77.50 | 3.79 | 5.287 |
|  |  |  | ± | 0.02 | 1.18 | 0.10 | 0.383 |
|  |  |  |  |  |  |  |  |
| MAG30c | 8 | 110 |  | 7.56 | 46.87 | 3.26 | 3.633 |
|  |  |  | ± | 0.02 | 2.80 | 0.09 | 0.318 |
|  |  |  |  |  |  |  |  |
| MAG31c | 8 | 120 |  | 4.72 | 33.44 | 4.02 | 2.887 |
|  |  |  | ± | 0.02 | 0.54 | 1.06 | 0.291 |
| MAG28c | 8 | 120-130 |  | 8.43 | 60.24 | 3.38 | 4.365 |
|  |  |  | ± | 0.02 | 3.59 | 0.09 | 0.371 |
|  |  |  |  |  |  |  |  |
| MAG52b | 12 | 130-140 |  | 8.19 | 61.73 | 3.71 | 4.480 |
|  |  |  | ± | 0.02 | 1.01 | 0.10 | 0.350 |
| MAG51b | 12 | 130-140 |  | 4.38 | 44.27 | 3.66 | 3.276 |
|  |  |  | ± | 0.02 | 0.76 | 0.10 | 0.291 |
|  |  |  |  |  |  |  |  |
| MAG34g | 8 | 145 |  | 7.58 | 54.49 | 3.78 | 4.099 |
|  |  |  | ± | 0.02 | 0.47 | 1.01 | 0.298 |
|  |  |  |  |  |  |  |  |
| MAG53b | 12 | 170-180 |  | 10.82 | 33.30 | 3.52 | 3.391 |
|  |  |  | ± | 0.02 | 0.54 | 0.09 | 0.288 |
|  |  |  |  |  |  |  |  |
| MAG33 | Bedrock |  |  | 3.53 | 37.43 | 4.02 | 2.952 |
|  |  |  | ± | 0.02 | 0.61 | 1.00 | 0.284 |
|  |  |  |  |  |  |  |  |
| MAG46c | Rock |  |  | 4.38 | 32.11 | 4.20 | 2.830 |
|  |  |  |  | 0.02 | 0.53 | 1.01 | 0.250 |
